# Wholesome Lunch to the Whole Classroom: Short‐ and Longer‐Term Effects on Early Teenagers' Weight

**DOI:** 10.1002/hec.4959

**Published:** 2025-03-18

**Authors:** Shiko Maruyama, Sayaka Nakamura

**Affiliations:** ^1^ Institute for Economic and Social Research Jinan University Guangzhou China; ^2^ Department of Economics Sophia University Tokyo Japan

**Keywords:** child obesity, difference‐in‐differences, heterogeneous treatment effect, school lunch, school nutrition programs

## Abstract

Previous studies on the effect of school lunch programs on child obesity have been hampered by effect heterogeneity, self‐selection, and stigma‐induced under‐reporting, having produced mixed findings. Its potential long‐lasting effect has also been debated. We study the body‐weight effect of a Japanese school lunch program, which provides nutritional lunch to *all* students at participating municipal junior highs. The lack of means testing and individual participation choice offers causal estimates of actual participation for a diverse and representative group of children. By exploiting almost universal school lunch coverage for elementary school children nationwide, we construct a difference‐in‐differences (DID) framework. Using the 1975–1994 National Nutrition Survey, a nationally representative household survey with measured height and weight, we find a regressive benefit of school lunch: while no statistically significant effect is found for the full sample, we find significant obesity‐reducing effects for the subsamples of children with low socioeconomic backgrounds. This obesity‐reducing effect remains at least a few years after graduation, implying effect through not only nutritional contents but also guiding healthy eating behavior. We find little evidence that school lunch reduces underweight. Propensity score weighting, the DID analysis for percentiles, and various falsification tests confirm the robustness of our estimates.

## 1 Introduction

Child obesity is a pressing public health concern, rapidly growing worldwide (Reilly and Kelly 2011; Ng et al. 2014). To combat this issue, school lunch reforms have recently been implemented. For instance, stricter nutritional standards were introduced in the UK and the US during the 2000s and 2010s, respectively, alongside the continuing expansion of eligibility for free school lunches in the UK (Evans and Harper 2009; Baidal and Taveras 2014). These reforms, however, have sparked heated debates due to their high costs (Tickle 2014, may 20; Baidal and Taveras 2014). Numerous studies have assessed the effect of school meal programs on body weight, but the findings have been inconsistent.^1^

An important, yet often overlooked aspect of school meal programs is their potential long‐lasting impact on obesity through shaping children's food preferences and eating habits. Nutrition education and promotion of healthy eating behaviors are the stated objectives of school lunch programs in many countries, including Italy, Sweden, Finland (Andersen et al. 2017), Japan (NIEPR 2013), and South Korea (Jo 2014). If a long‐lasting effect exists, it could substantially enhance a school meal program's cost‐effectiveness.

In this paper, we investigate the body‐weight effect of a Japanese school lunch program for students at municipal junior highs (public lower secondary schools for 12‐ to 15‐year‐olds). Unlike previously studied school meal programs, the Japanese program mandates *all students* at schools participating in the program to eat the provided lunch. We employ individual‐level data from the 1975–1994 National Nutrition Survey (NNS) to estimate the program's effect on weight‐for‐height, obesity, and underweight measures, including body mass index (BMI), and investigate how the effects vary by students' socioeconomic status. Furthermore, we estimate the program's lasting effects after students graduate from junior highs.

The unique features of the Japanese program enable us to advance the literature in several ways. Most importantly, we provide more credible and interpretable causal estimates than previous studies. The lack of consensus on school meal program effectiveness may partly stem from their voluntary participation. To address selection bias concerns, existing studies employ regression discontinuity design (RDD) based on eligibility cutoffs for school lunch subsidies (Schanzenbach 2009; Caro 2023) and difference‐in‐differences (DID) analysis based on policy changes that increase school lunch costs for households in specific income brackets (von Hinke Kessler Scholder 2013). Consequently, these estimates are a local average treatment effect (LATE) for specific income/SES groups affected by policy discontinuity or policy change. Furthermore, these estimates are interpreted as “intention to treat” (ITT) effects due to voluntary participation. While LATE parameters are informative for marginal policy changes, their external validity is not guaranteed. In the context of school lunch programs, local effects may vary substantially because the causal effect depends on not only what students eat at school lunch but also what they would have eaten without the program. School lunch will increase the weight of students who eat little without school lunch and decrease the weight of students who regularly consume energy‐dense diets such as French fries and sweetened beverages.

In contrast, the Japanese school lunch is communal, that is, all children attending schools providing school lunch must eat the provided lunch. This lack of individual choice in school lunch participation allows us to (1) estimate the effect of actual participation in the school lunch program (as opposed to an ITT effect) and (2) infer the overall program effect for a more diverse and representative group of children than previous LATE estimates.

Other distinctive features of the Japanese program allow us to obtain more interpretable estimates. First, strict national nutritional standards set by the government standardize the program and minimize regional differences across the country. These strict standards also make our estimates relevant and informative for policymakers in other countries where nutritional requirements are currently debated, as the literature has mainly focused on programs providing meals of questionable nutritional value. Second, there are no other large‐scale food provision programs in Japan. In the US, various means‐tested food assistance programs, such as the National School Lunch Program (NSLP), National School Breakfast Program, and the Supplemental Nutrition Assistance Program, coexist, complicating the analysis (Capogrossi and You 2017). Third, the non‐means‐tested nature of the program implies no stigma‐induced reporting bias in school lunch participation, contrasting with programs in many other countries. Gundersen et al. (2012) show that NSLP participation is significantly underreported, an issue overlooked in other studies.

We also advance the literature by examining effect heterogeneity by socioeconomic status (SES), an aspect few studies have addressed. Two recent US studies examine income‐related heterogeneity in the effect of programs that provide free meals to all students at participating schools: the Breakfast After the Bell program (Abouk and Adams 2022) and the Universal Free Meals Program (Schwartz and Rothbart 2020). However, students attending the participating schools predominantly come from low‐income families in both studies. In contrast, the Japanese setting allows us to examine effect heterogeneity across nearly the entire distribution of SES, as the Japanese program covers the majority of children in compulsory education without targeting either low‐income children or schools with predominantly low‐income students. Understanding individual‐level effect heterogeneity helps policymakers design effective and efficient school meal programs.

Lastly, this paper is among the few that study the persistent effect of school meals on weight. Past findings are mixed: Peterson (2014) reports a significant, positive association between adulthood obesity and NSLP participation, whereas Hinrichs (2010) finds no significant effect of NSLP on adulthood weight. Lundborg et al. (2022) study a universal school lunch program at Swedish elementary schools and find a significant positive effect on adulthood income and health. They find no effect on adult obesity; however, their study concerns the program's effect half a century ago when obesity prevalence was low and child malnutrition was a major issue.

Our identification strategy hinges on the variation in school lunch availability across municipalities. However, our estimate may be confounded if municipalities' decisions to provide school lunch reflect unobserved area characteristics. To address this concern, we employ a DID framework, exploiting elementary school children as pre‐treatment data. Japanese compulsory education comprises 6 years of elementary school for 6‐ to 12‐year‐olds and 3 years of junior‐high school for 12‐ to 15‐year‐olds, with the majority attending municipal schools (MEXT 2018). In contrast to substantial variation in municipal school lunch provision for junior highs, nearly all municipal elementary schools provide school lunch (NIEPR 2013). Leveraging this structure, we construct a DID framework in which we compare differences between junior‐high students (12–15 years old) and older elementary students (9–12 years old) across municipalities with and without school lunch provision for junior highs. Because our data does not follow the same municipalities or individuals over time, we cannot perform a conventional DID analysis exploiting the timing of municipality‐level policy changes in school lunch provision. Consequently, instead of the group and time fixed effects commonly used in standard two‐way fixed‐effect models, we employ group and age fixed effects to conduct a DID analysis on our data. We bolster our DID's common trend assumption by selecting treatment and control municipalities that are similar through DID estimation combined with propensity‐score trimming and inverse probability of treatment weighting (IPTW). We further apply our DID analysis to selected percentiles to examine how the distribution of weight measures varies by school lunch provision status.

While we find no significant effect of school lunch on body weight for the entire sample, we find a significant and sizable obesity‐reducing effect for the subsample of children with low socioeconomic backgrounds, with a weight loss of approximately 0.8–1 kg. We find little evidence that school lunch reduces underweight prevalence. The DID analysis for percentiles indicates that school lunch predominantly influences the higher quantiles of weight distribution. The weight‐reduction effect for low‐SES children persists several years after they graduate from junior high in a similar magnitude. To our knowledge, this study is the first to find the persistent weight‐reduction effect of school lunch. Our findings suggest that the effect operates not only through nutritional content but also by promoting healthy eating behaviors, providing evidence in favor of the cost‐effectiveness of the Japanese school lunch program as an obesity‐reduction intervention.

## 2 Backgrounds

School meal programs exhibit considerable variation across countries. Historically, originating from the charitable provision of free lunch for impoverished and undernourished schoolchildren in the mid‐19th century in the UK and the US. By the mid‐20th century, national‐level school lunch programs existed in Finland, France, Italy, Ireland, Portugal, Spain, and Sweden, focusing on increasing energy intake rather than reducing it (Evans and Harper 2009; Rutledge 2015). Currently, developed countries demonstrate substantial differences in the nutritional content, targeting, and policy objectives of school meal programs (Harper et al. 2008).

Japan started a universal and communal school lunch program that provides healthy meals nationwide in the 1950s, much earlier than other countries. As a program with these characteristics the Japanese school lunch program is still among the few and the largest in the world, covering the majority of students in compulsory education (Harper et al. 2008; NIEPR 2013).

The landscape of school lunch in Japan differs from that of many Western countries. In municipal elementary schools and junior highs, students are divided into classes of approximately 40 and spend the entire school day with their classmates, primarily at assigned seats in the homeroom. Students eat lunch with their classmates in the classroom. Except for a few schools offering menu choices, all students receive the same menu (Sanborn 2017). A typical menu comprises a meat or fish dish, vegetables, rice or bread, fruit, and milk (Nozue 2011). Designated students serve the meal. They are instructed to distribute food evenly among their classmates, though they may have additional servings if leftovers are available (Nozue 2011). Students eat together, and the homeroom teacher eats the same school lunch with the students and is expected to encourage students to eat everything served to them (NIEPR 2013). See Supporting Information S1: Appendix 1 for photos of typical lunch scenes at junior highs.

The first government‐subsidized school lunch program in Japan commenced in 1932 for elementary school children from low‐income families but was interrupted in 1944 due to World War II. The program was then resumed in 1946 in municipal elementary schools in Tokyo to combat child malnutrition resulting from severe food shortages. The program gradually expanded nationwide. The nutritional and sanitary guidelines became the School Lunch Law (henceforth SLL) in 1954, which was later revised to include municipal junior highs in 1956 (NIEPR 2013). The SLL mandates municipalities only to “make efforts” to provide lunch at municipal elementary schools and junior highs.

**FIGURE 1:**
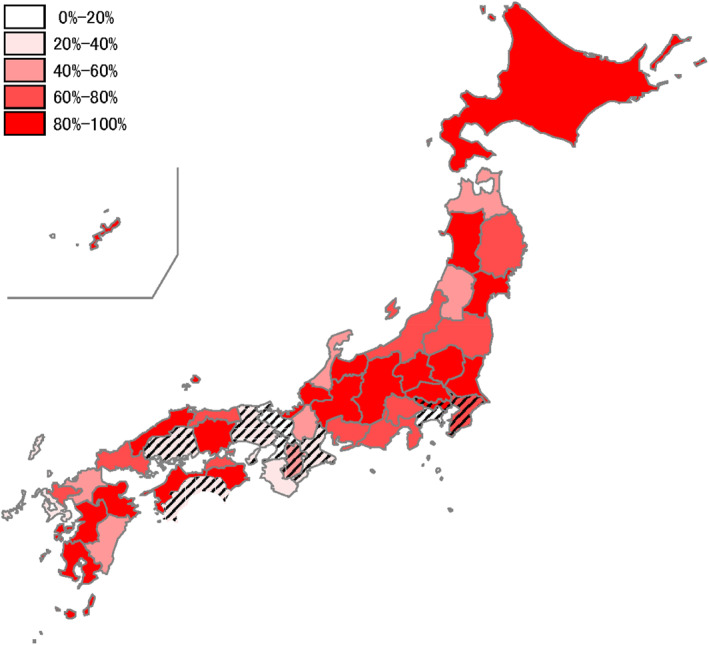
School lunch coverage rate for municipal junior‐high students in 1985 by prefecture. Shaded areas indicate the prefectures excluded from our sample. *Source:* National School Health Center of Japan (1986).

Since the late 1970s, the school lunch coverage rate for municipal elementary school students has remained above 98%, while the rate for municipal junior‐high students has increased more gradually, from 58% in 1978 to 83% in 2015, as detailed in Supporting Information S1: Appendix 1 (Figure A2). Figure 1 displays the 1985 school lunch coverage rate among municipal junior‐high students across prefectures, with even distribution throughout Japan and moderate spatial correlation.

The Ministry of Education^2^ specifies school lunch as part of the curriculum and requires all students to eat the provided school lunch, rather than bring their own food, if their school receives government funding for school lunch. The only exception to this requirement is for students with special dietary requirements, such as food allergies (Ono 2007). Financial constraints are unlikely to hinder children's participation because municipalities receive subsidies that cover the costs of construction and maintenance of facilities and the labor involved in food preparation. Children's guardians only pay ingredient costs and energy bills (NIEPR 2013). In 2018, for example, the average junior‐high lunch fee was 4941 yen (approximately 45 USD) per student per month (MEXT 2019). Furthermore, fees are waived for households receiving public assistance, and almost all municipalities provide school lunch to all students, regardless of fee payment (Fujisawa 2008). These compulsory and non‐means‐tested aspects of the Japanese school lunch program offer a unique opportunity to evaluate the direct effect of school lunch on a diverse group of children, rather than a LATE or ITT effect.^3^ Although the Japanese school lunch program has been criticized for its high costs and inefficiencies (Hironaka and Kasai 2019), our calculations indicate the program's cost‐efficiency, as discussed in Supporting Information S1: Appendix 1.

The Ministry of Education has set nutritional standards for school lunch, including target values for energy, protein, total fat, calcium, and vitamins since 1954 (Nozue 2011). During our study period, the standards underwent only one minor revision (see Supporting Information S1: Appendix 1). Japan's standards are significantly higher than their contemporary counterparts in the US and the UK. The current US federal requirements lack target values for protein, total fat, calcium, and vitamins, and only a minimum requirement for energy intake existed until 2012 (Baidal and Taveras 2014). In the UK, requirements for energy, protein, and fat for school lunch were introduced in 1966 but were abolished in 1980, leaving no legally binding nutrition requirements until 2000 (Evans and Harper 2009). In Japan, the Ministry of Education finds that actual intake from school lunch aligns well with the nutritional standards (National School Health Center of Japan 1990, 1991).

The SLL also stipulates *educational goals* for the school lunch program such as encouraging good eating habits, imparting knowledge on nutrition, and fostering sociability. The school lunch program has been part of the curriculum since 1958, instructing teachers to guide table manners and encourage healthy eating (NIEPR 2013). Since 2005, the school lunch program has been embedded as part of the national food and nutrition education program called *shokuiku*, which aims to promote healthy eating and prevent obesity and underweight.^4^

Despite the low average BMI of the Japanese, the prevalence of obesity‐related diseases in Japan is close to that of other developed countries (Guariguata et al. 2014). Because the rise in obesity‐related health risks begins at a lower BMI level among Asians than Caucasians, the Japan Society for the Study of Obesity has advocated defining obesity as a BMI of 25 or over since 2000, as opposed to the WHO's benchmark of BMI 30. Under this criterion, about 20% of adult Japanese have been obese since the 1950s (Kanazawa et al. 2002), raising concerns about child obesity, which is a major risk factor for adult obesity. BMI and obesity among Japanese children have significantly increased since the late 1970s (Yoshinaga et al. 2010; Maruyama and Nakamura 2015).

Underweight is also a serious public health issue among Japanese women. Female BMI has consistently decreased in post‐war Japan (Maruyama and Nakamura 2015). During our study period, the rate of 20‐ to 24‐year‐old women with a BMI of 18.5 or less increased from 15.8% to 23.2% (Takimoto et al. 2004).

We are aware of only one study on the effect of the Japanese school lunch program on child weight. Applying a fixed‐effects OLS model to 2006–2015 prefecture‐level data, Miyawaki et al. (2019) find a negative effect of the junior‐high school lunch coverage rate on overweight prevalence for boys only. They find no effect on underweight prevalence for either gender. However, the lack of time‐variant control variables and the use of lagged dependent variables make their OLS estimates less precise (Angrist and Pischke 2009, 243–246).^5^

## 3 Framework for Causal Analysis

### 3.1 Difference‐In‐Differences (DID) Analysis

We conduct DID analysis using data of elementary and junior‐high students drawn from the 1975–1994 NNS. Encrypted identifiers enable us to group residents by census district, a subarea of a municipality (henceforth “district”). Because a district is always within a municipality, the school lunch provision status should not vary within each district. As detailed in the next section, our data is repeated cross‐sections, not a panel, because the NNS does not allow us to follow the same districts or individuals over time. Consequently, instead of the district and time fixed effects commonly used in standard two‐way fixed effect models, we employ a DID framework with district and *age* fixed effects.

Without loss of generality, we regard having school lunch as the baseline case and assess the effect of *not* having school lunch for ease of presentation. To motivate our DID analysis, we begin with the following (non‐DID) linear regression for a sample of junior‐high students (not including elementary students):

(1) $$Y_{iad}=X_{iad}\beta +Z_{d}\gamma +\theta {\text{NoSchoolLunch}}_{d}+\alpha_{a}+\epsilon_{iad},$$

where $Y_{iad}$ denotes an outcome variable (e.g., BMI) of student *i* in age group *a* in district *d*, $X_{iad}$ is a vector of student *i*'s characteristics, including a constant term, $Z_{d}$ is a vector of district *d*'s characteristics, including dummies for prefectures and survey years, and $\alpha_{a}$ denotes age fixed effects. Because we do not track the same district or student over time, we suppress year subscripts for conciseness. ${\text{NoSchoolLunch}}_{d}$ is an indicator for no junior‐high school lunch in district *d*. Because each district is observed only once in our data, ${\text{NoSchoolLunch}}_{d}$ never varies within a district in our sample despite the gradual increase in school lunch provision at junior highs over time. $\epsilon_{iad}$ is the error term, and β and γ are parameters to be estimated. The “no school lunch” effect, θ in Equation (1), could be biased due to systematic differences in unobserved local obesogenic environments between districts with and without school lunch, such as access to healthy and unhealthy food, urban sprawl, access to parks and sports facilities, and transportation systems (Lake and Townshend 2006).

This bias could be addressed using a conventional DID analysis if we had municipality‐level panel data that allowed us to exploit the over‐time variation in school lunch status of each municipality. Unfortunately, we cannot follow the same municipality over time in our data (see Supporting Information S1: Appendix 2). Similarly, a DID analysis comparing over‐time changes in body weight between children with and without school lunch in junior‐high years is infeasible because we cannot follow the same children over time.

**FIGURE 2:**
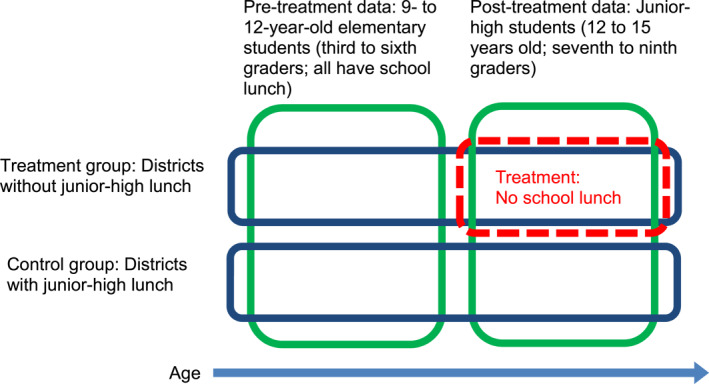
Illustration of the DID framework. In the Japanese compulsory education system, first to sixth graders attend elementary schools and seventh to ninth graders attend junior highs. Because the NNS is conducted in November each year, third to sixth graders in our sample are 9–12 years old, and the seventh to ninth graders are 12–15 years old. See Supporting Information S1: Appendix 2 (Figure A3) for the relationship among the school type, school grade, and age.

Instead, we capitalize on a unique feature of the Japanese school lunch program: nearly all municipal elementary schools provide school lunch. This allows us to form a DID framework in which we subtract the pre‐treatment difference, that is, the difference in outcome measures of elementary students between districts with and without junior‐high lunch, from the post‐treatment difference, that is, the same difference of junior‐high students, in order to adjust for the pre‐existing age‐invariant difference between the treatment and control districts, as illustrated in Figure 2. Under the age‐grade system in Japan, a child's school grade is determined strictly by the child's age on April 2nd. For elementary students, we limit our sample to 9‐ to 12‐year‐olds (Grades 3–6) for a better comparison with junior‐high students (Grades 7–9).

We estimate the following DID equation using the sample of elementary and junior‐high students:

(2) $$Y_{iad}=X_{iad}\beta +{\gamma \text{NoSchoolLunch}}_{d}+\theta {\text{JuniorHigh}}_{iad}\times {\text{NoSchoolLunch}}_{d}+\mu_{d}+\alpha_{a}+\epsilon_{iad},$$

where ${\text{JuniorHigh}}_{iad}$ is an indicator for junior‐high students, $\mu_{d}$ denotes district fixed effects, and θ is the DID estimator of the effect of not having school lunch. Because we now have district fixed effects and each district appears only once in the data, Equation (2) no longer has $Z_{d}$, the vector of district *d*'s characteristics, such as dummies for prefectures and survey years. Also, year subscripts are suppressed to make the notation concise. The JuniorHigh indicator appears only in the interaction term because junior‐high attendance is absorbed by age fixed effects $\alpha_{a}.$ This DID model uses age (denoted by subscript *a*) instead of time (commonly denoted by subscript *t*) in conventional DID models and is identified under the standard common trend assumption for $\epsilon_{iad}$ over age between the treatment and control districts, conditional on $X_{iad}$.^6^ We use standard errors clustered at the district level to allow for within‐district correlation of $\epsilon_{iad}$.^7^ Unlike prior quasi‐experimental studies, we quantify the effect of *actual* participation rather than the “intention to treat” effect.

Our DID is a variant of the two‐way fixed effect models widely used in the context of DID analysis. Because the treatment varies with age but not with time, we use age and group fixed effects instead of the time and group fixed effects commonly employed in conventional DID models. Our identification does not rely on within‐group changes in the outcome variables over time, in common with cohort DID models developed by Duflo (2001) and sibling/household fixed effects models used extensively in the literature (e.g., Chen et al. 2024; Pollakowski et al. 2022).

The recent two‐way fixed‐effect literature has indicated difficulties in interpreting the estimate under different treatment timings (e.g., staggered adoption) and effect heterogeneity among groups (Callaway and Sant’Anna 2021; de Chaisemartin and D’Haultfoeuille 2020). However, this is not an issue in our study because no staggered policy changes are used. In our DID analysis, age replaces time, and the treatment always begins and ends at the same ages for every student.

### 3.2 Subsample Analysis

We examine effect heterogeneity for low‐SES children, a group of policy interest for two reasons. First, in many countries, including the UK, the US, and Canada, the primary target of school meal programs is children in low‐income families (Harper et al. 2008). Second, we expect larger effects for lower‐SES children. Low parental SES is associated with children's poor diet quality (Darmon and Drewnowski 2008), less healthy eating habits (Hazano et al. 2017), and obesity (Shrewsbury and Wardle 2008; Lee 2013; Kachi et al. 2015). T. A. Smith (2017) finds a larger impact of the US school meal programs on children's nutrition for children with poorer diet quality. Similarly, Japanese studies find a smaller household income gradient in nutritional quality among elementary students on days with school lunch than days without (Yamaguchi et al. 2018; Horikawa et al. 2020; Murayama et al. 2021).

We conduct a subsample analysis for two low‐SES groups. Because our data lack information on parental education or income, we first use paternal occupations as proxies for SES. We study a subsample of children whose fathers are not in white‐collar occupations, that is, those whose fathers are non‐white‐collar workers or not employed (“children with non‐white‐collar fathers,” henceforth). We focus on the father's occupation due to the strong persistence of male breadwinner families, with a small women's share of income in married couples (Kohara 2007) and lower labor participation among wives with higher‐income husbands (Higuchi 1995). See Supporting Information S1: Appendix 2 for further details, including our definition of white‐collar workers.

The actual SES of these households, however, may change over time, affecting comparability across children of different ages and potentially causing bias in the estimated longer‐term effects. To address this concern, we study another subsample: children in households with per‐member expenditure below the median (“children with low household expenditure,” henceforth).^8^ Household expenditure is a reliable measure of SES, capturing material well‐being for low‐income households (Meyer and Sullivan 2003). In addition to the median, we repeat the analysis using other percentiles.

### 3.3 Inverse Probability of Treatment Weighting (IPTW) and Propensity‐Score Trimming

As a robustness check, we use IPTW and propensity‐score trimming in the DID analysis (henceforth, DID‐IPTW). Combining DID analysis with propensity score methods effectively eliminate potential sources of temporally invariant bias (J. A. Smith and Todd 2005), balancing baseline characteristics between the treatment and control groups and making the common trend assumption more plausible. We first estimate the propensity score of *not* having junior‐high lunch for each district and then conduct propensity‐score trimming, which excludes districts with extreme propensity scores to ensure sufficient overlap in characteristics between the treatment and control districts. Finally, we assign an IPTW weight equal to the inverse of the propensity score of the child's realized treatment status to each child in the trimmed sample. Under a set of assumptions, these weights, called ATE weights, allow us to consistently estimate the average treatment effect (ATE) (Imbens 2004). The IPTW method can also identify the average treatment effect on the treated (ATT) using different weights (ATT weights). Results with ATT weights are discussed in Subsection 6.3. See Supporting Information S1: Appendix 3 for details on the IPTW and trimming.

## 4 Data

### 4.1 The National Nutritional Survey

We use a sample of children aged 9–15 in elementary and junior‐high schools drawn from the 1975–1994 NNS. The NNS is a nationally representative, cross‐sectional survey conducted annually by the Ministry of Welfare.^9^ Katanoda et al. (2005) confirm the NNS's representativeness. The response rate is reasonably high: among approximately 5000 households requested to participate in the 2002 survey, 4160 participated (MHLW 2003). The survey includes information on body measurements, diet, and sociodemographic characteristics of each household member. Height and weight are measured without shoes and adjusted for the weight of clothes by health professionals, ensuring accuracy and eliminating self‐reporting bias (Gorber et al. 2007). Details of the sample construction are provided in Supporting Information S1: Appendix 2.

### 4.2 Municipal Provision of School Lunch

We impute the school lunch provision status of each district using NNS information on whether a child eats school lunch. In the 1975–1994 NNS, each participating household is requested to choose three consecutive days excluding Sundays and holidays and report the details of all meals each member had each day. We assume that school lunch is provided if it is reported for at least one of the 3 days.

Individual reports might not accurately reflect the municipal school lunch status, even for weekdays, for two reasons. First, some children might miss school lunch due to absence. Second, because the NNS contains no information on the school children attend, school lunch status can be misclassified if children choose to attend private or national schools. To address the second classification error, we exclude 10 prefectures where more than 5% of junior‐high students attended non‐municipal schools in 1994 (shown as shaded areas in Figure 1).

Our sample occasionally exhibits disagreement within a district, in which case we determine the school lunch status based on the majority rule: whether 50% or more of junior‐high students report having school lunch. To improve accuracy, we exclude districts with only one junior‐high respondent and districts with exactly two conflicting answers. Additionally, when the school lunch provision status is evident from official statistics, we use that information instead of survey responses. Details on the procedure to determine the school lunch status are provided in Supporting Information S1: Appendix 2. In Supporting Information S1: Appendix 2, we also confirm that the derived school lunch status is highly accurate and consistent with the official statistics. To address remaining concerns regarding the imputation procedure, we confirm the robustness of our main findings to the exclusion of districts with mixed reports on junior‐high lunch (See Subsection 6.3).

Our final sample comprises 8482 junior‐high students and 9823 elementary students from 2271 districts, of which 500 districts do not provide school lunch in their municipal junior highs.

### 4.3 Weight Measures, Control Variables, and Summary Statistics

Our outcome variables include BMI, defined as (weight in kilograms)/(height in meters),^2^ binary indicators for obesity and underweight, and another measure of weight‐for‐height widely used in Japan called percentage of overweight (POW). POW is the proportion of weight deviation to the standard weight‐for‐height by age and gender, defined as ([weight]–[standard weight])/(standard weight). We obtain standard weight‐for‐height for Japanese children from Murata and Itoh (2003). We also examine height in some analyses. For BMI and height, we use both the raw value and the *z*‐score, normalized by gender, age, and 5‐year cohort.

Defining obesity and underweight requires caution because children's BMI distribution varies substantially by age, gender, and race. We use the extended International Obesity Task Force (IOTF) definition (Cole and Lobstein 2012) and the POW definition. The IOTF definition is a BMI‐based definition widely used to account for racial differences in growth patterns. The cutoff for obesity for each age‐gender group is determined based on the percentage rank corresponding to BMI 30.0 at age 17.5. We use BMI 25.0 for the obesity cutoff instead of the widely‐used 30.0, following the recommendation by the Japan Society for the Study of Obesity (Kanazawa et al. 2002). Underweight is defined analogously: the percentage rank corresponding to BMI 18.5 at age 17.5 is used to determine the underweight cutoff. These IOTF cutoffs minimize across‐age variation in the prevalence of obesity and underweight, thus fitting well within our DID framework. For Asian children, the IOTF definition is preferred over the WHO reference, which is based on American children's BMI distribution (de Wilde et al. 2013). We use the gender‐ and age‐specific BMI distributions for Japanese children reported in Kato et al. (2011).

Under the POW definition, children whose weight exceeds their standard weight by 20% (i.e., POW > 20%) are categorized as obese. The POW definition has an advantage over BMI‐based measures because BMI increases with height for children in puberty (Sugiura and Murata 2011).

Table 1 shows the summary statistics of height and weight measures of elementary and junior‐high students by treatment status for the full sample (Panel [a]) and the two low‐SES subsamples (Panels [b] and [c]). Junior‐high students have larger BMIs than elementary students, but the other weight measures show no clear age trends, as expected. The two obesity measures are reasonably consistent. Children in the two socioeconomically disadvantaged subsamples are consistently shorter than children in the full sample, but the weight measures show no clear difference. In all three samples, comparison between the treatment and control groups among elementary students shows little difference, except that BMI, POW, and obesity prevalence are smaller in the treatment districts than in the control districts. In contrast, among junior‐high students, these weight and obesity measures are all larger in the treatment districts than in the control districts in all three samples, consistent with the formal DID results below.

**TABLE 1:** Summary statistics: Outcome variables.

	Elementary students				Junior‐high students			
	Districts with no junior‐high lunch		Control districts		Districts with no junior‐high lunch		Control districts	
	Mean	Std. dev.	Mean	Std. dev.	Mean	Std. dev.	Mean	Std. dev.
(a) Full sample								
Height (cm)	138.544	8.387	138.450	8.547	158.306	7.656	158.064	7.624
Height in *z*‐score	0.034	0.963	−0.031	0.983	0.052	0.969	−0.018	0.981
BMI	17.169	2.397	17.304	2.475	19.662	2.748	19.629	2.646
BMI in *z*‐score	−0.033	0.929	−0.008	0.958	0.030	1.006	−0.004	0.956
POW	−0.608	12.778	0.287	13.150	−0.047	13.580	−0.359	12.896
Obese (IOTF)	0.069	0.253	0.077	0.267	0.063	0.243	0.058	0.235
Obese (POW)	0.069	0.253	0.079	0.270	0.071	0.257	0.066	0.248
Underweight (IOTF)	0.159	0.365	0.152	0.359	0.159	0.366	0.154	0.361
Number of children	2088		7735		1780		6702	
Number of districts	500		1771		500		1771	
(b) Children with non‐white‐collar fathers								
Height (cm)	137.993	8.297	138.280	8.520	157.858	7.405	157.649	7.640
Height in *z*‐score	−0.030	0.951	−0.051	0.990	0.023	0.941	−0.069	0.999
BMI	17.091	2.365	17.361	2.530	19.800	2.815	19.628	2.684
BMI in *z*‐score	−0.052	0.905	0.023	0.987	0.092	1.034	0.001	0.972
POW	−0.609	12.922	0.780	13.477	0.696	13.953	−0.253	13.024
Obese (IOTF)	0.067	0.250	0.082	0.275	0.073	0.261	0.056	0.229
Obese (POW)	0.071	0.257	0.084	0.278	0.082	0.274	0.064	0.244
Underweight (IOTF)	0.165	0.372	0.145	0.352	0.147	0.354	0.155	0.362
Number of children	1017		3887		858		3420	
Number of districts	349		1305		349		1305	
(c) Children with low household expenditure								
Height (cm)	138.471	8.521	138.221	8.677	157.890	7.630	157.785	7.633
Height in *z*‐score	−0.005	1.010	−0.077	0.984	−0.016	0.961	−0.062	0.996
BMI	17.075	2.213	17.298	2.520	19.712	2.905	19.682	2.686
BMI in *z*‐score	−0.080	0.868	−0.010	0.978	0.048	1.055	0.020	0.964
POW	−1.002	11.806	0.459	13.277	0.253	14.354	0.005	12.949
Obese (IOTF)	0.057	0.232	0.079	0.270	0.075	0.263	0.058	0.234
Obese (POW)	0.056	0.230	0.082	0.274	0.086	0.280	0.063	0.244
Underweight (IOTF)	0.162	0.369	0.155	0.362	0.165	0.371	0.150	0.357
Number of children	825		3680		734		3150	
Number of districts	289		1166		289		1166	

To enhance the credibility of our DID estimates, we include controls for various child, parental, and household characteristics to account for differential age trends specific to each area, which cannot be addressed by district fixed effects. For child characteristics, we use gender‐specific age dummies, with separate age dummies for 12‐year‐old elementary students and 12‐year‐old junior‐high students. Parental characteristics comprise age, height, BMI, and occupation dummies of the father and mother. Household characteristics include the coresidence of father, grandfather, and grandmother; the number of children in the household; and the percentage ranking of monthly household expenditure per household member.

Table 2 reports the summary statistics of the control variables by treatment status with the normalized difference between the two groups.^10^ The table shows that junior‐high lunch is more likely to be provided in districts where children live with their grandparents, parents work in agriculture/fisheries/forestry, and per‐member expenditure is lower. The absolute normalized differences in the control variables between the treatment and control groups are overall small. IPTW and propensity‐score trimming make these differences even smaller, as shown in Supporting Information S1: Appendix 3 (Table A3).

**TABLE 2:** Summary statistics: Control variables.

	Districts with no junior‐high lunch		Control districts		
Variable	Mean	Std. dev.	Mean	Std. dev.	Normalized difference
Male	0.521	0.500	0.515	0.500	0.013
Age	11.763	1.998	11.811	2.010	−0.024
Father’s age	38.151	13.043	38.082	12.945	0.005
Father’s height (*z* score by age, sex, and 5‐year cohort)	0.024	0.851	−0.038	0.844	0.072
Father’s BMI (*z* score by age, sex, and 5‐year cohort)	−0.017	0.864	0.014	0.846	−0.035
Father’s height and BMI missing	0.256	0.437	0.252	0.434	0.010
Father: White‐collar worker (the reference category)	0.360	0.479	0.346	0.475	0.029
Father: Laborer	0.296	0.455	0.292	0.453	0.009
Father: Self‐employed	0.188	0.390	0.182	0.385	0.016
Father: Agriculture/fisheries/forestry	0.053	0.222	0.077	0.265	−0.102
Father: Other occupation (not working)	0.009	0.088	0.009	0.092	−0.003
Without father in household	0.094	0.292	0.093	0.291	0.003
Mother’s age	39.552	4.328	39.553	4.406	0.000
Mother’s height (*z* score by age, sex, and 5‐year cohort)	−0.019	0.980	−0.073	0.974	0.056
Mother’s BMI (*z* score by age, sex, and 5‐year cohort)	−0.001	0.926	0.093	0.997	−0.097
Mother’s height and BMI missing	0.037	0.188	0.037	0.188	0.000
Mother: White‐collar worker (the reference category)	0.170	0.372	0.161	0.364	0.023
Mother: Laborer	0.236	0.421	0.246	0.427	−0.022
Mother: Self‐employed	0.137	0.343	0.130	0.334	0.023
Mother: Agriculture/fisheries/forestry	0.055	0.226	0.094	0.288	−0.150
Mother: Other occupation (not working)	0.401	0.485	0.369	0.478	0.066
Grandfather in household	0.165	0.371	0.204	0.403	−0.100
Grandmother in household	0.272	0.445	0.316	0.465	−0.096
# of children in household (below 18 years old)	2.276	0.813	2.310	0.777	−0.042
Per‐member household expenditure ranking (defined between 0.0 and 1.0 where 0.0 means households with highest expenditures in each survey year)	0.607	0.239	0.643	0.241	−0.149
Number of children	3868		14,437		
Number of districts	500		1771		

*Note:* The last column shows the normalized difference between the treatment and control groups. All statistics are based on non‐missing observations. When body measurement data (height and BMI) are missing, dummies for the missing values are used in the regression analysis. Because the household expenditure is reported in intervals that vary by survey year, we construct the percentage rank within all households in each survey year for comparability across years. The ranking is recorded in descending order: a larger value implies a smaller expenditure.

### 4.4 Determinants of School Lunch Provision

Table 2 indicates that the junior‐high lunch status is not purely random. Even if we control for observable district characteristics, the unconfoundedness assumption might be violated. Our DID estimation addresses this non‐randomness and yields unbiased causal estimates under the common trend assumption. Specifically, the assumption requires that the school lunch provision be determined orthogonally to children's growth in height and weight from age 9 to 15 (after controlling for characteristics that vary over age).

To assess this premise, it is helpful to understand the determinants of school lunch provision at junior highs. We first conduct a district‐level Logit regression analysis in which we regress the *NoSchoolLunch* dummy on the means of body measurement variables (i.e., mean height, mean BMI, the prevalence rates of obesity and underweight) among children aged 1 to 11, and other district characteristics, as detailed in Supporting Information S1: Appendix 4. The regression estimates indicate lower junior‐high lunch coverage in larger municipalities and a significant increase in school lunch provision over time, consistent with both the nationwide and sample trends. On the other hand, we find no other strong determinants of municipal junior high lunch provision. Most importantly, the body measurement variables of elementary and preschool children are independently and jointly nonsignificant, suggesting that the municipal school lunch provision is unrelated to the stature, obesity, and underweight of children. The lack of significance of the expenditure level implies that the municipal decision on school lunch is not driven by either the economic deprivation of residents or the municipality's budget availability. Occupation is a major determinant of obesogenic environments (Maruyama and Nakamura 2018), but occupational composition variables are not significant. These findings suggest the absence of a direct link between the school lunch provision and obesogenic environments around children, supporting our common trend assumption.^11^

Our DID framework requires a common trend over age in the other determinants of body weight besides school lunch between the treatment and control groups. The lower coverage of junior‐high lunch in more urban areas and in earlier survey years raises concerns that the trend of the physical activity level over age might vary by urbanicity and survey year. Because the NNS contains little information on children's physical activity levels, we examine the physical fitness test score of municipal elementary and junior‐high students as a proxy for their physical activity level using aggregate data retrieved from the Physical Fitness and Athletic Performance Survey conducted by the Ministry of Education. We find little variation in the age trends by urbanicity and no consistent chronological trends in the score by age and gender, which does not support such concerns, as detailed in Supporting Information S1: Appendix 4.

A strong correlation of school lunch provision within a prefecture may mislead the statistical inference because our analysis assumes random sampling at the district level. Further data analysis shows that this is not a major concern, as detailed in Supporting Information S1: Appendix 1.

## 5 Results

### 5.1 Main Results

**FIGURE 3:**
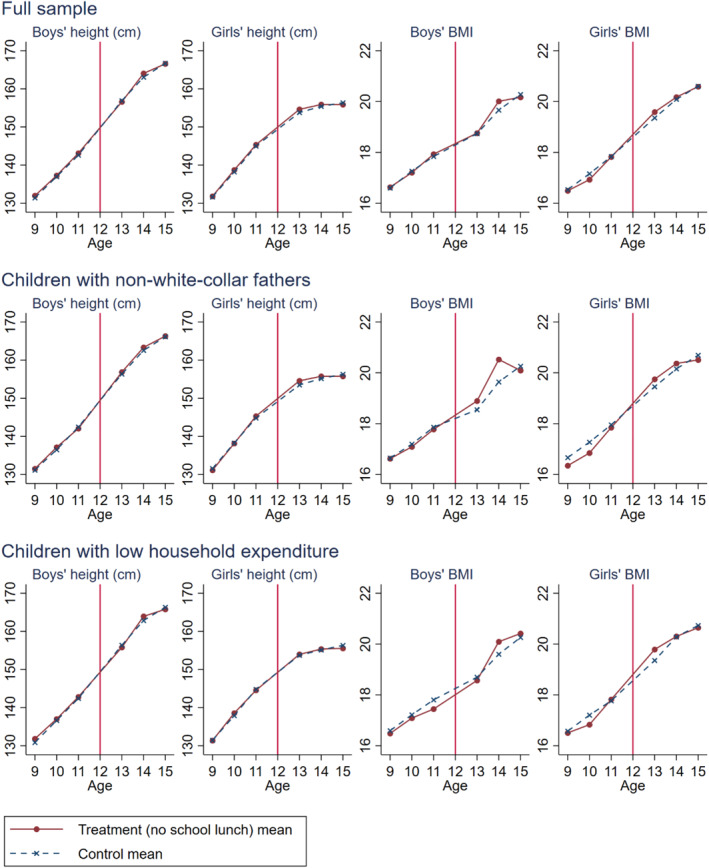
Means of height and BMI across age. The lines plot year‐adjusted means. To adjust for time trends and over‐time changes in survey‐year composition, each year's value is adjusted by the deviation from the aggregate mean. Values for 12‐year‐olds are omitted because our sample excludes them for 1975–1985 due to data limitations.

Figure 3 plots year‐adjusted means of height and BMI by gender in the full sample and the low‐SES subsamples over ages 9 to 15 for the treatment and control groups. The vertical red line indicates age 12, the threshold age between elementary and junior‐high students. In this figure, 15‐year‐olds include both current‐ and post‐junior‐high students. For all three samples, the trends in mean height are highly similar between the treatment and control groups for both genders, highlighting little difference in growth patterns, and we further confirm this in subsection 6.2. The differences in BMI trends are also small in the full sample. Among children with non‐white‐collar fathers, elementary boys' BMI is similar between the groups and elementary girls' BMI is lower for the treatment than the control groups, whereas both genders' BMI of the treatment group exceeds that of the control group at ages 13 and 14. Among children with low household expenditure, fluctuations in BMI are large, but the overall trends are similar to those of children with non‐white‐collar fathers.

Table 3 reports the estimated effects of no school lunch from (a) junior‐high OLS, as specified in Equation (1), (b) DID regression, and (c) DID‐IPTW regression in Panels (a)–(c), respectively.^12^ While we find no statistically significant effect on any outcome measure across all three specifications except for POW in the base DID for the full sample, the subsample results reveal robust evidence that school lunch reduces BMI, POW, and obesity for children from lower socioeconomic backgrounds. In the subsample of children with non‐white‐collar fathers, the estimated effects are significantly positive on BMI, BMI *z*‐score, POW, and both obesity measures in all three specifications, at least at the 10% level. In the subsample of children with low household expenditure, while none of the estimated effects from the OLS are significant except for the obesity status under POW definition, those from the base DID and DID‐IPTW are all significantly positive on all outcomes except for the underweight measure. For both low‐SES subsamples, the estimated effects on weight measures and obesity status are all larger with trimming and IPTW. We find no evidence that school lunch affects underweight prevalence in any specification regardless of the sample used. We also conduct a subsample analysis of children whose per‐member household expenditure is *above* the median and children with fathers in white‐collar occupations or without fathers, and none of the estimated effects are significant.^13^

**TABLE 3:** Effects of no school lunch.

Sample	# Children	# Districts	BMI	BMI *z*‐score	POW			Obesity (IOTF)	Obesity (POW)	Underweight (IOTF)
Panel (a): Junior‐high OLS										
Full sample	8482	2271	0.043 (0.076)	0.019 (0.028)	0.153 (0.385)			0.000 (0.007)	0.002 (0.008)	0.003 (0.011)
Children with non‐white‐collar fathers	4278	1654	0.232** (0.111)	0.088** (0.042)	0.990* (0.565)			0.020** (0.010)	0.021* (0.011)	−0.015 (0.015)
Children with low household expenditure	3884	1455	0.031 (0.124)	0.022 (0.046)	0.122 (0.626)			0.012 (0.011)	0.020* (0.012)	0.023 (0.018)
Panel (b): Base DID										
Full sample	18,305	2271	0.102 (0.091)	0.036 (0.035)		0.811* (0.471)	0.008 (0.009)		0.008 (0.010)	0.001 (0.014)
Children with non‐white‐collar fathers	9182	1654	0.428*** (0.137)	0.163*** (0.052)		2.224*** (0.716)	0.034** (0.014)		0.029** (0.014)	−0.028 (0.020)
Children with low household expenditure	8389	1455	0.332** (0.153)	0.126** (0.057)		2.067*** (0.793)	0.046*** (0.015)		0.053*** (0.015)	0.001 (0.022)
Panel (c): DID‐IPTW										
Full sample	13,985	1741	0.092 (0.110)	0.038 (0.041)		0.748 (0.561)	0.002 (0.011)		0.007 (0.011)	−0.000 (0.017)
Children with non‐white‐collar fathers	6512	1173	0.536*** (0.177)	0.207*** (0.067)		2.822*** (0.909)	0.040** (0.017)		0.045** (0.018)	−0.026 (0.023)
Children with low household expenditure	6462	1128	0.410** (0.165)	0.168*** (0.061)		2.440*** (0.843)	0.052*** (0.017)		0.056*** (0.017)	0.001 (0.025)

*Note:* Standard errors clustered at the district level are in parentheses. For the list of control variables in each regression, see Table 2. OLS in Panel (a) also controls for the NNS‐based district characteristics listed in Supporting Information S1: Table A6.

*, **, and *** indicate statistical significance at the 10%, 5%, and 1% levels, respectively.

We can convert these estimates to a weight change, assuming a junior‐high student with the mean height in each subsample. For children with non‐white‐collar fathers, the estimated effects on BMI and POW both imply a weight gain of about 1.1 kg. For children with low household expenditure, the estimated effect on BMI implies a weight gain of about 0.8 kg and that on POW implies a gain of about 1.0 kg.

### 5.2 DID for Percentiles

**FIGURE 4:**
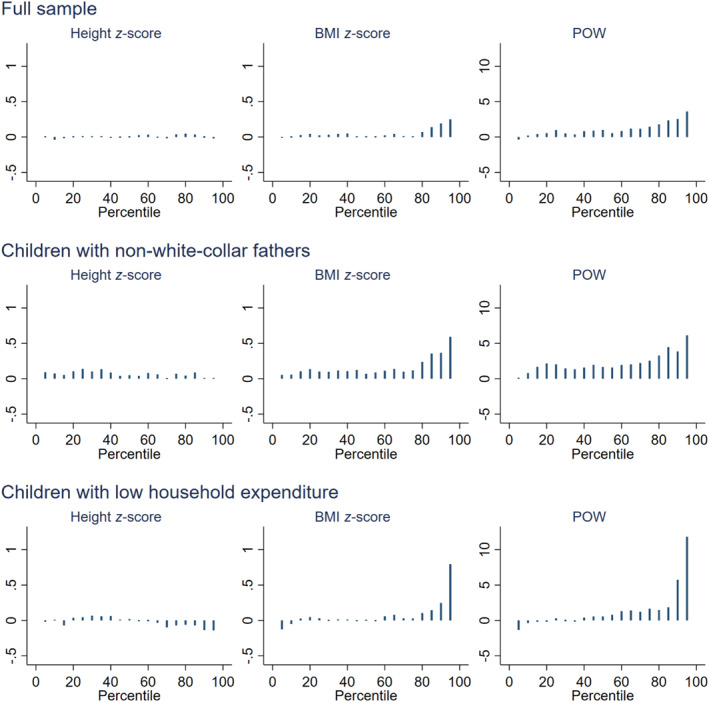
DID estimates of the effect of no school lunch for junior‐high students using percentiles. The point estimates, shown as bars, are obtained by calculating the difference of differences with respect to each of the 19 equidistant intervals between the 5th and 95th percentiles.

To explore how the distribution of outcome variables is affected by the school lunch program, we conduct a DID analysis for percentiles and take the double differences of corresponding percentiles instead of means. Specifically, for each of the treatment and control groups, we calculate the difference of $Y_{id}$ at a certain percentile between junior‐high students and elementary students. We then subtract the calculated difference for the control group from that for the treatment group to obtain the DID estimate.^14^ Figure 4 shows the point estimates for height in *z*‐score, BMI in *z*‐score, and POW. For all three samples, the estimates for height are close to zero across quantiles. In contrast, the estimates for BMI and POW are mostly positive and larger for higher quantiles for all three samples, with more substantial changes for the low‐SES subsamples than for the full sample.

We avoid making a strong rank‐invariance assumption, necessary to interpret the results as an individual‐level treatment effect. Figure 4 indicates an outward expansion in the right tail of the distribution of weight measures for the treatment group relative to the control group (school lunch shrinks the right tail of the distribution). Without the rank‐invariance assumption, this larger weight reduction effect is driven by either formerly obese children or formerly nonobese children with a high risk of rapid weight gain. We do not speculate which scenario is at work. In either case, our DID result shows that school lunch reduces both the prevalence of heavy weight and disparity in body weight.

### 5.3 Additional Subsample Analyses

To further explore effect heterogeneity by household expenditure, we repeat the main analysis using different thresholds to define the group of children with low household expenditure. As expected, the weight reduction effect is larger for children with lower household expenditures (Supporting Information S1: Appendix 4).

We also conduct two more sets of subsample analysis to explore the mechanisms behind the main results. The first is a subsample analysis of children whose mothers have high BMI. Maternal overweight is a strong predictor of their children's overweight. Potential mechanisms include genetic transmission, transmission of obesogenic habits, and shared environment (Dolton and Xiao 2017). These imply a larger weight reduction effect of school lunch for children with high‐BMI mothers. Second, we conduct a subsample analysis of children living in districts with high energy intake, using information on household‐level energy intake in the NNS. Higher energy intake may reflect more obesogenic local food environment or residents' stronger preference for high‐caloric meals, and these may lead to a larger weight reduction effect of school lunch.^15^ The results are reported in Table 4. Consistent with our conjectures, the results show evidence of obesity reduction effects among children with above the 75th percentile maternal BMI and among children with above the 75th percentile district‐level energy intake. These results, together with the results of our main analysis (Table 3) and DID for percentiles (Figure 4), indicate a significant weight reduction effect of school lunch for children at risk of high energy intake.

**TABLE 4:** Effects of no school lunch: Children at risk of high energy intake.

Sample	# Children	# Districts	BMI	BMI *z*‐score	POW	Obesity (IOTF)	Obesity (POW)	Underweight (IOTF)
Panel (a): DID								
Maternal BMI: Above median	7827	1583	0.186 (0.153)	0.065 (0.058)	1.212 (0.779)	0.008 (0.017)	0.011 (0.018)	−0.005 (0.019)
Maternal BMI: Above 75 percentile	3191	876	0.347 (0.226)	0.132 (0.087)	2.015* (1.181)	0.050* (0.027)	0.050* (0.027)	−0.022 (0.028)
District energy intake level: Above median	8935	1122	0.186 (0.123)	0.076 (0.047)	1.041 (0.636)	0.017 (0.012)	0.014 (0.013)	−0.016 (0.019)
District energy intake level: Above 75 percentile	4349	540	0.312* (0.170)	0.130** (0.064)	1.669* (0.878)	0.037** (0.017)	0.038** (0.018)	−0.025 (0.026)
Panel (b): DID‐IPTW								
Maternal BMI: Above median	5778	1181	0.286 (0.185)	0.108 (0.070)	1.596* (0.935)	0.014 (0.021)	0.004 (0.021)	−0.023 (0.022)
Maternal BMI: Above 75 percentile	2407	669	0.621** (0.270)	0.243** (0.104)	3.505** (1.447)	0.078** (0.032)	0.063** (0.031)	−0.043 (0.032)
District energy intake level: Above median	7467	919	0.181 (0.138)	0.071 (0.052)	0.918 (0.715)	0.025* (0.013)	0.021 (0.013)	−0.009 (0.022)
District energy intake level: Above 75 percentile	4054	494	0.252 (0.182)	0.107 (0.069)	1.278 (0.953)	0.039** (0.018)	0.035* (0.019)	−0.028 (0.028)

*Note:* Standard errors clustered at the district level are in parentheses. For the list of control variables included in each regression, see Table 2.

*, **, and *** indicate statistical significance at the 10%, 5%, and 1% levels, respectively.

### 5.4 The Cost‐Effectiveness of the Japanese School Lunch Program

We attempt a back‐of‐the‐envelope cost‐effectiveness analysis focusing on the obesity reduction effect and abstracting away from other benefits. As presented in Supporting Information S1: Appendix 4 in detail, the estimated cost per obesity case prevented is comparable to the corresponding estimates of other countries' programs.

## 6 Robustness Tests

In subsection 4.4, we have discussed the policy exogeneity. In this section, we further examine the internal validity of our analysis in various ways: a permutation test, a falsification test, and an event study analysis. We also address potential confounding by heterogenous effects of attending junior high, explore effect heterogeneity, and implement a synthetic control method. Lastly, we examine the robustness of our findings to an alternative weighting method and sample specifications.

### 6.1 Permutation Test

To assess the potential underestimation of standard errors due to correlation of the error terms among children close in age and location, we implement permutation tests in the spirit of Bertrand et al. (2004) and Abadie et al. (2010). As detailed in Supporting Information S1: Appendix 5 (Figure A6), the implied statistical significance in this test is highly consistent with the corresponding results in Table 3, suggesting that the statistical significance of our estimated school lunch effects is not due to the misspecification of correlation structure.

### 6.2 Falsification Test: Regression Analysis of Height

Our DID framework relies on the common trend assumption that the school lunch provision is determined orthogonally to children's growth in height and weight from age 9 to 15 (after controlling for covariates that vary over age). This assumption might be violated if there are systematic differences in the growth pattern between the treatment and control districts, especially in the timing of puberty onset. While IPTW, propensity‐score trimming, and the use of POW should mitigate the potential bias, we further conduct a falsification test similar to Cawley et al. (2013) and Havnes and Mogstad (2011), in which we use height instead of weight measures as a regressand, as detailed in Supporting Information S1: Appendix 5. Because height is determined primarily by genetic factors and early‐life environment, and the effect of adolescent lifestyle is limited (Beard and Blaser 2002), school lunch should not have a strong, immediate effect on height. None of the DID estimates are significant for both the full sample and the low‐SES subsamples, providing additional support for the common trend assumption.

### 6.3 Event Study Analysis

To further examine whether our results are driven by pre‐existing differential growth patterns between the treatment and control groups, we extend our baseline model by interacting the *NoSchoolLunch* dummy with age dummies and explore how the DID estimator evolves with children's age. As detailed in Supporting Information S1: Appendix 5 (Figure A7), we find no evidence of significant pre‐existing differential trends.^16^

### 6.4 Other Robustness Checks

Further robustness checks support the robustness of our results.^17^ Our results might be confounded by the differential effects of attending junior high by urbanicity and survey timing. To address this concern, we add interactions of the *JuniorHigh* dummy with 5‐year period dummies, prefectural population density, and municipality size variables to the DID regression model. Furthermore, we also test specifications with triple interactions of the *JuniorHigh* dummy, year dummies, and prefecture dummies to the DID regression model to allow for differential effects of attending junior high by prefecture‐year. Our results are robust to these modifications.^18^

To examine possible heterogeneity of the effect of school lunch by time period and urbanicity, we conduct subsample analysis by time period and subsample analysis by urbanicity. We do not find strong evidence of treatment heterogeneity by time period or urbanicity.

We implement a synthetic control method proposed by Abadie et al. (2010) to compare the outcome trends of the treatment group with an artificial control group exhibiting more similar pre‐treatment trends than the original control group. The results confirm the robustness of our findings.

The DID with IPTW reported in Table 3 employs ATE weights, while the IPTW method can also recover ATT using ATT weights. Using ATT weights in place of ATE weights, we find little difference, implying no evidence of large effect heterogeneity between the treatment and control districts, as shown in Supporting Information S1: Appendix 3.

Further robustness tests regarding sample construction are conducted. First, we relax the exclusion criterion of the prefectures by lowering the required share of junior‐high students attending municipal schools in 1994 from 95% to 90%. This modification does not change our findings substantially. The results remain largely unchanged even when we use all 47 prefectures. Second, we estimate the same model excluding 12‐ and 15‐year‐olds. Among 12‐year‐olds, some are elementary and others are junior‐high students, depending on the birth month. Similarly, some of the 15‐year‐olds are current junior‐high students and others have finished junior high. Hence, our results might be affected by potential differential effects of the birth month on children's height and weight between the treatment and control groups. We confirm the robustness of our findings to this exclusion and also find highly similar effects of the birth month on height for both groups. Third, as detailed in Subsection 4.2, for districts with mixed reports on junior‐high lunch we determine the school lunch status based on the majority rule. To address concerns about misclassification in this procedure, we repeat the main analysis excluding these districts and confirm that our findings do not change significantly.

## 7 Longer‐Term Effects

Does the weight reduction effect for low‐SES children persist after they graduate from junior high and are no longer subject to the school lunch program? The persistent effect of school lunch is a parameter of policy interest because it can drastically alter the cost‐benefit of such programs. The literature suggests that it takes a relatively short time for body weight to adjust to an increase in energy balance (Hall et al. 2011). This implies that the direct effect of school lunch wanes shortly after children graduate from junior high, and hence any longer‐term effect should be attributed to indirect effects. While we refrain from speculating underlying mechanisms, indirect effects may arise via changes in food preferences, eating habits, and enhanced food knowledge. School lunch may induce students to habituate to healthy and balanced food in appropriate amounts and to develop an ability to choose such food. Eating habits are persistent and have a strong impact on food intake (Atkin 2016), and adolescence is a critical period for the formation of obesogenic eating styles (Alberga et al. 2012).

To estimate the longer‐term effect of school lunch, we exploit the fact that the Japanese school meal program does not cover post‐junior‐high schools.^19^By adding 15‐ to 17‐year‐old post‐junior‐high students to the sample, we estimate the following model:

(3) $$Y_{id}=X_{id}\beta +{\gamma \text{NoSchoolLunch}}_{d}+\theta^{S}{\text{JuniorHigh}}_{id}\times {\text{NoSchoolLunch}}_{d}+\;\theta^{L}{\text{PostJH}}_{id}\times {\text{NoSchoolLunch}}_{d}+\mu_{d}+\epsilon_{id},$$

where ${\text{PostJH}}_{id}$ is an indicator for post‐junior‐high students, and $\theta^{S}$ and $\theta^{L}$ denote the DID estimators of short‐ and longer‐term effects of “no school lunch,” respectively.^20^

Table 5 reports estimated $\theta^{S}$ and $\theta^{L}$. As expected, the estimated effects for junior‐high students (henceforth “short‐term effects”) closely align with the corresponding results in Table 3. They are all nonsignificant except for POW in the base DID for the full sample, and all significantly positive for the low‐SES subsamples. The estimated longer‐term effects for post‐junior‐high students are all positive. At the 10% significance level, only two out of 10 estimates of the longer‐term effects are significant for the full sample, while nine out of 10 are significant for children with non‐white‐collar fathers, and eight out of 10 are significant for children with low household expenditure. For the low‐SES subsamples, many of the estimated longer‐term effects demonstrate a similar effect size as the short‐term effects.

**TABLE 5:** Short‐ and longer‐term effects of no school lunch.

			BMI	BMI *z*‐score	POW	Obesity (IOTF)	Obesity (POW)
Panel (a): Base DID							
Full sample (23,472 children, 2265 districts)		NoSchoolLunch × JuniorHigh	0.094 (0.089)	0.032 (0.034)	0.798* (0.462)	0.007 (0.009)	0.008 (0.010)
		NoSchoolLunch × PostJH	0.025 (0.102)	0.002 (0.040)	0.505 (0.523)	0.006 (0.011)	0.010 (0.011)
Children with non‐white‐collar fathers (11,744 children, 1657 districts)		NoSchoolLunch × JuniorHigh	0.401*** (0.134)	0.153*** (0.051)	2.136*** (0.699)	0.033** (0.013)	0.029** (0.014)
		NoSchoolLunch × PostJH	0.280* (0.153)	0.100* (0.058)	1.663** (0.770)	0.033** (0.016)	0.026 (0.016)
Children with low household expenditure (10,534 children, 1547 districts)		NoSchoolLunch × JuniorHigh	0.355** (0.152)	0.137** (0.057)	2.135*** (0.783)	0.046*** (0.015)	0.053*** (0.015)
		NoSchoolLunch × PostJH	0.342** (0.162)	0.138** (0.063)	2.113*** (0.818)	0.026 (0.018)	0.031* (0.017)
Panel (b): DID‐IPTW							
Full sample (17,987 children, 1729 districts)	NoSchoolLunch × JuniorHigh		0.044 (0.109)	0.021 (0.041)	0.568 (0.557)	−0.001 (0.011)	0.004 (0.011)
	NoSchoolLunch × PostJH		0.146 (0.130)	0.062 (0.050)	1.201* (0.665)	0.010 (0.013)	0.022* (0.013)
Children with non‐white‐collar fathers (8105 children, 1121 districts)	NoSchoolLunch × JuniorHigh		0.486*** (0.172)	0.188*** (0.065)	2.548*** (0.887)	0.036** (0.017)	0.039** (0.018)
	NoSchoolLunch × PostJH		0.447** (0.197)	0.176** (0.076)	2.518** (0.985)	0.036* (0.020)	0.037* (0.019)
Children with low household expenditure (7571 children, 1119 districts)	NoSchoolLunch × JuniorHigh		0.351** (0.164)	0.143** (0.062)	2.118** (0.831)	0.042** (0.017)	0.050*** (0.017)
	NoSchoolLunch × PostJH		0.508*** (0.183)	0.212*** (0.072)	2.818*** (0.928)	0.032 (0.022)	0.042** (0.019)

*Note:* PostJH is a dummy variable for 15‐ to 17‐year‐old students who are not junior‐high students. Standard errors clustered at the district level are in parentheses. For the list of control variables included in each regression, see Table 2.

*, **, and *** indicate statistical significance at the 10%, 5%, and 1% levels, respectively.

These findings provide suggestive evidence that the obesity‐reducing effect of school lunch for low‐SES children persists at least several years after they graduate, consistent with the Japanese government's policy to promote healthy eating habits and food knowledge through school lunch. These results also offer favorable evidence regarding the cost‐effectiveness of the Japanese school lunch program as an obesity‐reduction intervention and suggest that when the persistent effects are disregarded, as is the case with our cost‐effectiveness calculation above, this may lead to substantial underestimation of the program's effectiveness.

## 8 Conclusion and Discussion

We examine the causal effect of the Japanese school lunch program on the weight of junior‐high students using data drawn from the 1975–1994 NNS. To account for possible endogeneity of the municipal provision of school lunch, we employ a DID framework that compares differences between elementary and junior‐high students across districts with and without school lunch at junior highs. The results show that the lack of school lunch significantly increases the BMI and obesity rate of children from low socioeconomic backgrounds. We find little evidence that school lunch affects underweight prevalence, implying the variance‐reduction effect of school lunch. The obesity‐reducing effect of school lunch for low‐SES children appears to last at least several years after graduation, suggesting that the school lunch effect involves not only direct changes in caloric and nutritional intake but also changes in behavior.

This study has the following policy implications. First, school lunch with strict nutritional requirements can reduce child obesity. This finding is consistent with recent findings that the Chilean School Meals Program reduces obesity partly via improvements in nutritional quality (Caro 2023) and that provision of nutritious school lunch reduces obesity in England (Holford and Rabe 2022). Second, the compulsory nature of the Japanese school lunch program, with its primary purpose of abolishing stigma, might play a key role in obesity reduction because children with a strong preference for fattening food might avoid school lunch if a choice is allowed. Third, school lunch might have long‐lasting obesity‐reducing effects via preference and habit formation, consistent with the educational goals of the Japanese school lunch program of promoting good eating habits and food knowledge (NIEPR 2013). Fourth, the obesity reduction effects of school lunch for low‐SES children might have contributed to the absence of significant income gradient in child obesity among elementary‐school students (Kachi et al. 2015) and the small income gradient in child health in Japan (Nakamura 2014).

We find an obesity reduction effect of school lunch only for children with low‐SES backgrounds. This suggests that, from obesity reduction perspectives, the main target of school lunch should be children from low‐income families and overweight children. Policy options include an optional school lunch program with subsidies for these targeted groups. However, the Japanese universal and compulsory school lunch program has several possible advantages over a more targeted program. First, children with strong preferences toward fatty food might avoid school lunch even if school lunch is available at a low out‐of‐pocket cost, and it might be difficult to make school lunch compulsory only for children with particular characteristics. Second, universal school lunch might increase positive peer effects from classmates with healthy eating habits and facilitate food education. Third, if the government subsidizes school lunch only for children from low‐income families and overweight children, that could produce stigma (Altindag et al. 2020). Stigma could not only hurt children's self‐esteem and trigger bullying and discrimination but also reduce program participation, as is the case with the Japanese public assistance program (Inaba 2011).

## Conflicts of Interest

The authors declare no conflicts of interest.

## Supporting information

Supporting Information S1

## Data Availability

The dataset used in the main analysis was drawn from microdata accessible only by approved registered users; hence, we have no discretion to provide the microdata for public use and must request an exemption from the journal's data policy. However, the data are available to other researchers through the standard application scheme.

## Endnotes

^1^
von Hinke Kessler Scholder (2013) finds the UK school lunch program has had no significant effect on child body weight in the 1980s, whereas Holford and Rabe (2022) find obesity reduction effects of the recent universalization of the English school lunch program. For the US National School Lunch Program, some studies find weight‐increasing effects (Schanzenbach 2009; Li and Hooker 2010; Millimet et al. 2010; Hernandez et al. 2011; Capogrossi and You 2017), but others find no significant effect (Hofferth and Curtin 2005; Gleason and Dodd 2009; Mirtcheva and Powell 2013) or do find weight‐reduction effects (Gundersen et al. 2012). For the US National School Breakfast Program, some studies find weight‐reduction effects (Gleason and Dodd 2009; Millimet et al. 2010; Millimet and Tchernis 2013), while others find no significant effects (Hofferth and Curtin 2005; Sudharsanan et al. 2016).

^2^
The Ministry of Education was consolidated into the Ministry of Education, Culture, Sports, Science, and Technology (MEXT) in 2001.

^3^
An increasing number of municipalities nowadays allow students to choose between school lunch and home‐prepared lunch, but such a choice was rare during our study period (see Supporting Information S1: Appendix 1).

^4^
The Third Basic Program for Shokuiku Promotion (In Japanese): www.mhlw.go.jp/file/06‐Seisakujouhou‐10900000‐Kenkoukyoku/0000129496.pdf (Accessed on September 18, 2021).

^5^
Other Japanese studies find a positive association between school lunch and the intake of vegetables and dairy products (Takahashi et al. 1983; Kawaraya and Mori 2009; Nozue et al. 2010). National School Health Center of Japan (1990) reports the poor nutritional content of school day lunch among junior high students without school lunch.

^6^
We require common trends over age within a survey year, whereas standard DID models based on individual‐ or municipality‐level panel data require common trends over age for a certain birth cohort or common trends over years for a certain age group.

^7^
We cluster standard errors at the district level and not the municipality level due to data limitations. However, this is unlikely to cause significant underestimation, because the probability that the NNS randomly samples multiple districts from the same municipality is negligibly rare.

^8^
See Supporting Information S1: Appendix 2 for the definition of the median per‐member household expenditure.

^9^
The NNS was renamed the National Health and Nutrition Survey in 2003. The Ministry of Welfare was consolidated into the MHLW in 2001.

^10^
For DID studies normalized differences are more effective for assessing the difficulty of adjusting for covariate differences between the groups than *t*‐statistics (Imbens 2015).

^11^
We also conduct substantial archival work, examining media coverage, research articles, and minutes of municipal assemblies, to understand the determinants of school lunch provision, but little information has been identified.

^12^
Full regression results are available from the authors upon request.

^13^
The results are available from the authors upon request.

^14^
We do not conduct formal quantile DID analysis and statistical inference because the econometric theory for the quantile treatment effect in DID settings with control variables is under development and because the proposed methods in recent studies are intended primarily for balanced panel data (Lamarche 2021) and do not seem to fit our two‐way fixed effects analysis using cross‐sectional data.

^15^
Details of the energy intake variable are provided in Supporting Information S1: Appendix 2.

^16^
This is not an event study model in a strict sense as the model uses age instead of time. Additionally, due to the difference between age and school grade, the age‐specific DID estimates for junior high students do not precisely capture the dynamic effect of the treatment.

^17^
The results are available upon request.

^18^
Another robustness test of the common trend assumption would be to include linear age trends specific to each district in the DID analysis. However, this is not appropriate in our setting because the growth curves of height and weight over age are highly nonlinear.

^19^
Exceptions are part‐time senior highs that a small fraction (2.7% in 1985) of senior high students attend and special‐needs schools (NIEPR 2013; MEXT 2018).

^20^
We cannot estimate longer‐term effects beyond the age of 18 because relocation of individuals is very common after 18.
