# Supplementary material for: Wholesome Lunch to the Whole Classroom: Short‐ and Longer‐Term Effects on Early Teenagers' Weight
Source: Health Econ. 2025 Mar 18;34(7):1255–73. doi: 10.1002/hec.4959 (PMC12166539; doi:10.1002/hec.4959)
Supplement: Supplementary file 1 — Supporting Information S1 [file HEC-34-1255-s001.pdf]

For Online Publication

Appendices to "Wholesome Lunch to the Whole Classroom: Short- and Longer-Term Effects on Early Teenagers' Weight"

Appendix 1. Details of the Japanese School Lunch Program

Typical lunch scenes at junior highs

Figure A1 shows typical lunch scenes at junior highs. As shown in Panel (a), designated students serve the meal. Students eat the same menu in the classroom together with their classmates, as shown in Panel (b).

Trends in School Lunch Provision

Official statistics on trends in school lunch provision are drawn from the School Lunch Data Book (SLDB) for 1978–2004 and the Current Status Survey on School Meals (CSSSM) for 2006–2015. The SLDB is published by the National School Health Center of Japan, and the CSSSM is conducted by the Ministry of Education, Culture, Sports, Science, and Technology (MEXT).[1] We calculate the annual nationwide school lunch coverage rate as a percentage of the total municipal school students at municipal schools with school lunch.

Municipalities provide either "complete school lunch," "complementary school lunch," or "milk-only school lunch." "Complementary school lunch" provides dishes except for staple foods, such as steamed rice or bread, which students bring themselves. "Milk-only school lunch" only provides milk, and students bring their food. We use the sum of "complete school lunch" and "complementary school lunch" to calculate the school lunch coverage rate, though the latter has always been less than one percent for both elementary and junior-high students.

[1] www.mext.go.jp/b_menu/toukei/chousa05/kyuushoku/1267027.htm (Accessed Sep. 18, 2021)

Figure A2 shows trends in the national school lunch coverage rate for municipal junior-high students and in the coverage rate in our sample (as defined in Subsection 4.2). Both lines show similar increasing trends, although the latter is always higher than the former. This difference can be explained by low school lunch coverage rates in prefectures excluded from our sample, as detailed in Appendix 2. The school lunch coverage rate for municipal elementary school students (not shown in the figure) has been above 98 percent since 1978.

Optional School Lunch Programs

Since the 1990s, the Ministry of Education has allowed municipalities to make their school lunch programs optional, as a temporary measure for schools that have newly started school lunch programs (Asahi Shimbun, 1996). Under an optional lunch program, students have a choice between a school lunch and a home-prepared lunch, which might hinder the estimation of causal effects of having school lunch on child outcomes. During our study period 1975–1994, however, only a handful of municipalities had optional programs, and we remove all children who could have optional school lunch from our sample, as detailed in Appendix 2.

Revisions in Nutritional Standards

Nutritional standards in the Japanese school lunch program have been revised eight times since 1954. During our study period (1975–1994), revision occurred only once in 1986. In the 1986 revision, the three-year age categories for elementary school children were replaced by the two-year categories; target values for fat (17g, 20g, and 24g for age groups 6–8, 9–11, and 12–14, respectively) were replaced with the maximum percentage of energy intake from fat of 30 percent; and the target amounts were slightly reduced for energy (e.g., from 850kcal to 820kcal for ages 12–14) and protein

(e.g., from 36g to 32g for ages 12–14) (Nozue, 2011). The revision did not cause significant changes in the energy or fat contained in school lunch (Narusaka, 1996).

Cost of the School Lunch Program

For the fiscal year of 2018, we estimate the mean public expenditure on school lunch as 426 yen (3.86 USD) and children's guardians' expenses to be 264 yen (2.39 USD) per meal, adding up to 690 yen (6.25 USD). Here, the mean annual public expenditure per meal is obtained by dividing the municipal expenditure on the costs of labor, property, and maintenance and repair of the property associated with school meals by the total number of school meals, following Nomura (2020). Data on municipal expenditure are retrieved from the Local Finance Statistics Annual Report conducted by the Local Public Finance Bureau, Ministry of Internal Affairs and Communications, and data on the number of school lunch meals and the school lunch fees are obtained from the Current Status Survey on School Meals conducted by MEXT.[2]

This total cost per meal is not insignificant and, for instance, exceeds the average amount college students spend for lunch at a university co-op cafeteria of 490 yen (4.44 USD) (National Federation of University Co-operative Associations, 2020). Indeed, the Japanese school lunch program has been criticized for its high cost and inefficiency in the procurement and preparation of school lunch, due to the lack of incentives in the public sector and MEXT's outdated regulations (Hironaka and Kasai, 2019). However, the opportunity cost of preparing a packed lunch for parents is also not negligible. According to a large-scale survey, adults spend 18 minutes on average to prepare a packed lunch (Nichirei Foods 2019), and the burden is borne predominantly by mothers.

[2] https://www.mext.go.jp/b_menu/toukei/chousa05/kyuushoku/1267027.htm (Accessed Sep. 18, 2021).

Even among senior high students, the majority (91.8 percent) have their parents prepare their packed lunch to bring to school (Norinchukin Bank, 2017), and in Japan husbands on average spend only 0.496 hours on household chores and childcare during workdays, whereas wives spend 6.681 hours (Sasaki 2018). Based on the mean female wage rate by five-year age groups in MHLW (2018), the time cost of 18 minutes amounts to 451–496 yen (4.08–4.49 USD) for 30- to 59-year-old women, exceeding the public expenditure per meal of 426 yen (3.86 USD), which covers labor, property, and maintenance and repair of the property associated with school meals.

Within-Prefecture and Spatial Correlation in Municipal Provision of School Lunch

Municipalities' school lunch status might be correlated within a prefecture, particularly due to the influence of the prefectural government. To examine the degree of within-prefecture variation in school lunch status, we adjust the *NoSchoolLunch* dummy by subtracting the annual prefectural school lunch non-coverage rate obtained from SLDB and then compare the all-sample standard deviations of *NoSchoolLunch* dummies with and without this adjustment. We find that the adjustment reduces the standard deviation only slightly, from 0.408 to 0.377, suggesting large within-prefecture variation and relatively small between-prefecture variation.

Spatial correlation of municipal school lunch provision might also arise from the reference behavior among neighboring municipalities and spatial correlation in unobservables. We cannot directly examine this possibility because data limitations prevent us from identifying the proximity of sampled municipalities. However, given the sampling scheme of the NNS, the probability that districts in the same or adjacent municipalities are sampled is fairly small. Even in 1976, the year when the NNS sample size is the largest, the NNS randomly sampled only about 11 percent of municipalities across the country.

Appendix 2. Details of Data Construction and Sample Selection

Difficulties in Conducting a Municipality-Level Panel Analysis

As explained in the paper, municipalities in our data are de-identified with a scrambled district ID. If there is only one municipality in a municipality size category in a prefecture, the municipality can be identified, but in our sample, this is the case for only three cities in the largest municipality size category: Sapporo, Sendai, and Nagoya. Moreover, in large cities like these, junior-high lunch was sometimes introduced at different times at different schools for various reasons, including a pilot program or municipal merger (Asahi Shimbun, 1993; City of Sapporo, 1997; Nagoya Narumi Junior High School, 2019), which makes a municipality-level panel analysis difficult. Note that the variation in adoption timing within a municipality is unlikely to affect our analysis because it will not cause within-district variation in school lunch status, as school districts closely overlap with census districts, the geographical observation unit in the NNS.

Definition of Non-White-Collar Fathers

We conduct a subsample analysis for children whose fathers are not in white-collar occupations, i.e., whose fathers are in non-white-collar occupations or not in employment (referred to as "children with non-white-collar fathers," in this paper). We define white-collar workers as white-collar employees (employed workers engaged in technical, educational, clerical, or managerial works), business owners not in technical occupations, and self-employed professionals (freelance workers engaged in works that require professional knowledge or expertise, such as practicing clinicians and lawyers). We call workers in all the other occupational categories non-white-collar workers, which are laborers (employees engaged in manual labor such as factory workers, drivers, sales clerks, and service workers), non-professional self-employed workers (owners and family employees of small business, including merchants and craftsmen), and workers in agriculture, fisheries, or forestry.

Definition of Median Per-member Household Expenditure

The median per-member household expenditure is defined among children of the same two-year age group in the same survey year. Because per-member household expenditure in the NNS is reported in intervals that vary by year, we cannot determine which children in the median interval are below the median. To address this, we rank children first by the interval and second by the district mean of the per-member household expenditure ranking (defined in Table 2) and use this rank to define the median.

Measures of Nutritional Intake and Protein Deficiency

In this subsection, we explain the compilation of nutritional intake data and its utilization for calculating protein deficiency prevalence and conducting subsample analyses by energy intake level. The NNS provides household-level nutritional intake information derived from a nutritionist-assisted food intake questionnaire. In the questionnaire, the household member responsible for cooking records all food items and their quantities consumed during the study period. The survey's purpose and procedure are thoroughly explained to participants, who are instructed to use scales for measurements. Additionally, certified nutritionists visit each household to offer further guidance and correct misreporting. Maruyama and Nakamura (2018) confirm the validity of the NNS food intake data.

We employ the household-level ratio of actual to required protein intake to examine protein deficiency levels among children in our sample. The robustness test in Subsection 6.2, which uses height as an outcome, instead of weight measurements, necessitates the assumption that junior-high school lunches have no effect on students' height growth. This assumption is violated if malnutrition-induced stunting occurs in children and school lunches improve nutrition, promoting height growth. Consistent with this possibility, Lundborg et al. (2021) find a positive impact of a 1960s Swedish elementary school lunch program rollout on adulthood height, attributing the results to reduced

protein deficiency since protein is crucial for height growth (Grasgruber et al., 2014). We examine protein intake levels to investigate this scenario's applicability to our sample. Based on Hosoya et al. (1985), we employ the third edition of the Recommended Dietary Allowance for Japanese (RDA-J) to determine protein requirements by age, gender, and pregnancy/breastfeeding status. For each household, we divide actual protein intake by the sum of household members' protein requirements. The sample mean of this ratio is 1.697 with a standard deviation of 0.318, suggesting that the majority have protein intake substantially exceeding the requirement. The proportion of children with household-level protein intakes below the requirement is 0.55 percent, suggesting minimal room for height increases through protein deficiency reduction.

Furthermore, we construct a measure of energy intake at the district level to conduct the subsample analysis of children living in districts with high energy intake, as described in Section 5.3. District-level energy intake is constructed as follows. First, we regress the household-level energy intake on household composition variables to predict energy intake for each household. Specifically, we categorize household members into age groups (1–2, 3–5, 6–7, 8–9, 10–11, 12–14, 15–17, 18–29, 30–49, 50–64, 65–74, 75 and older), height groups (ten groups of equal size for each gender), occupational groups (laborers; white-collar workers; agriculture/forestry/fishery; students in kindergarten and compulsory education; students in secondary or higher education; and homemakers/other occupations/occupations unknown), and by pregnancy (19 weeks or later) and breastfeeding status. The age categorization is based on that for the estimated energy requirement by gender in Dietary Reference Intakes for Japanese.[3] We then regress the household-level energy intake on year dummies and the number of household members in each of these categories by gender. We divide the sample into four five-year periods and separately predict the energy intake for

[3]https://www.mhlw.go.jp/stf/seisakunitsuite/bunya/kenkou_iryou/kenkou/eiyou/syokuji_kijyun.html (Accessed Aug. 12, 2021).

each sub-period. Second, we calculate the ratio of reported energy intake to predicted energy intake as a measure of the energy intake level for each household. Third, the district mean of this ratio is calculated as a measure of district-level energy intake. In calculating the mean, we exclude households with junior-high students because school lunch may affect the energy intake of these households. Households without members aged 59 or younger are also excluded from this calculation, as the food intake of these elderly households may substantially differ from households with elementary and junior-high students.

Identifying Elementary and Junior-High Students

Figure A3 presents the relationship among the school type, school grade, and age for children in our sample (9- to 15-year-olds in compulsory education). The NNS questionnaire asks if children are in compulsory education but does not distinguish between elementary schools and junior highs. Because a child's school grade in Japan is strictly determined by the child's age on April 2nd, we can use the children's age to categorize 6- to 11-year-old and 13- to 15-year-old children in compulsory education as elementary and junior-high students, respectively. In the 1975–1985 NNS, the school grade of 12-year-olds cannot be determined, but for those who are 12 years old in 1986–1994, we can use the birth month information. Because the NNS is conducted in November, we categorize 12-year-old children at the time of the survey as elementary students if they are born between April and October, and those born between December and March as junior-high students. We exclude 12-year-olds for 1975–1985 from the sample because we cannot determine their school grade. November-born 12-year-old children in the 1986–1994 surveys are also excluded for the same reason. While our method misclassifies first-year junior-high students born on April 1st as sixth-year elementary students, the effect of this misclassification is negligibly small.

Identifying Fathers and Mothers

Because the NNS does not record kinship among household members, we regard 27- to 59-year-olds in a child's household as parents and those 60 years old or older as grandparents. 5.4 percent of children have multiple "fathers" or multiple "mothers", and mean values are used in these cases.

Sample Exclusion Criteria

Our sample consists of 9- to 15-year-old children attending elementary schools and junior highs, excluding all 12-year-olds from the 1975–1985 NNS and November-born 12-year-olds from the 1986–1994 NNS, previously outlined. We exclude children in prefectures with a high proportion of junior-high students attending non-municipal schools and those in Nagoya city in 1993 and 1994, as detailed below. Next, we restrict our sample to children with valid height and weight information and exclude those with a *z*-score height exceeding an absolute value of 4.0, those with a POW of −45 percent or lower (i.e., weight is 55 percent or less than the standard weight-for-height), and those with a POW of 100 percent or higher (i.e., weight is double or more than the standard weight-for-height), as these values may reflect genetic growth disorders or data coding error. These POW thresholds follow medical guidelines for emergency hospitalization at −45 percent or lower (Suzuki, 2016) and metabolic surgery at 100 percent or higher (Kawamura, 1995). A small number of children lacking a mother in the household or valid household expenditure data are also excluded. We further refine our sample to include children in districts with at least one elementary student and one junior-high student, as required for DID. Finally, we exclude children in districts with unreliable school lunch information or where less than half of the elementary school children report having school lunch, as detailed below in this Appendix. Figure A4 presents changes in sample size after applying these exclusion criteria.

Reasons for Time Frame Restrictions

Although the NNS provides individual-level data annually since 1975, we do not use data collected after 1994 for two reasons. First, post-1994 food diaries only cover one day of each household's choice from weekdays and Saturdays. As our data lack information on the day of the week and school lunch is not served on Saturdays, our imputed school lunch status will be understated due to families choosing Saturday for the food diary. Second, the expansion of optional school lunch programs since the late 1990s complicates the causal interpretation of our estimate, as detailed in Appendix 1 and the subsequent subsection.

Exclusion of Municipalities with Optional School Lunch Programs

As explained in Appendix 1, municipalities initiating school lunch programs after the 1990s may opt to make their school lunch program optional, allowing students to choose between school lunch and home-prepared lunch (Asahi Shimbun, 1996). This is a potential concern for our analysis because optional programs might attenuate our treatment-effect estimate. Among municipalities in our data, Nagoya city launched an optional trial program at seven municipal junior highs in 1993 (Asahi Shimbun, 1993). We identify children living in Nagoya in 1993 and 1994 using prefecture and municipal population size information, and exclude them from our final sample. Funabashi and Matsudo cities in Chiba prefecture also began optional school lunch programs before 1994 (Asahi Shimbun, 1993), but this does not affect our sample because it does not include children in Chiba prefecture due to its high proportion of junior-high students attending non-municipal schools, as explained below.

Municipal Provision of School Lunch

The NNS samples districts from all Japanese prefectures and requests all households in the sampled districts to participate in the survey. Only an encrypted identifier of the district of residence and the name of the prefecture are provided in the publicly available NNS dataset; thus, we impute the school lunch provision status of each district using NNS information on whether a child eats school lunch.

This imputation involves several classification issues. The first issue is that school lunch is served only on weekdays, whereas the NNS does not specify which day of the week each food diary record refers to. We solve this problem by exploiting the unique feature of the 1975–1994 NNS: each participating household is requested to choose *three consecutive days* excluding Sundays and holidays and report the details of all meals each member had each day. This means that each household's survey period covers at least two consecutive weekdays. We assume that school lunch is provided if it is reported for at least one of the three days.

From individual reports, we can only determine whether a child had school lunch on specific days, and this information may not accurately reflect municipal school lunch provisions for several reasons. Firstly, some children may miss school lunch due to illness or extracurricular activities, such as field trips. Secondly, children might attend municipal schools outside their municipality. Thirdly, a considerable number of junior-high students attend private and national schools, and few of these schools provide school lunch. We presume that the first type of measurement error is minimal because we use a three-day food diary, and the NNS instructs households to select a survey period representative of their usual diet, avoiding periods with special events. The second type of measurement error is also expected to be small due to the strict Japanese school district system. Attending municipal schools outside of one's municipality is only permitted for special reasons, such as geographic difficulties in commuting to the designated school (Nakamura, 2000). Some parents may provide a false resident registration to enable their children to attend municipal schools outside their district (e.g., Mainichi, 2015), but this is illegal and rare.

To address the third type of measurement error, we exclude prefectures with a high proportion of junior-high students attending non-municipal schools. Since the NNS does not collect information on school ownership types, we identify such prefectures using the number of junior-high students by prefecture and ownership type from the School Basic Survey conducted by MEXT.[4] Considering the increasing proportion of junior-high students attending non-municipal schools over time, we exclude ten prefectures where the proportion was five percent or higher in 1994: Tokyo, Kochi, Nara, Kanagawa, Kyoto, Hyogo, Hiroshima, Osaka, Chiba, and Mie (listed in descending order of proportion). This exclusion eliminates 35.6 percent of the observations. The proportion of junior-high students attending non-municipal schools reached 5.7 percent at the national level in the last year of our study period, 1994, but excluding those prefectures reduces it to 2.6 percent. The proportion is even lower in earlier years.

We argue that our imputed school lunch status data are reasonably accurate due to the exclusion of these prefectures, the use of the majority rule, and the exclusion of districts with insufficient information. Additionally, we supplement our imputation with official statistics from the School Lunch Data Book (SLDB). Specifically, we consider children in prefectures with 99 percent or higher school lunch coverage, according to the SLDB, as having school lunch. For years without SLDB statistics, we apply the same imputation using linearly interpolated values. School lunch provision at a municipal elementary school is determined analogously.

To demonstrate the accuracy of the imputed school lunch status, Table A1 presents the district-level distribution of the percentage of students reporting having school lunch. In 2,219 districts (97.4 percent), the majority of elementary student respondents report having school lunch, aligning with the nearly 100 percent school lunch coverage rate in official reports (Appendix 1). For junior-high students, the reported share is one in 65.6 percent and zero in 20.6 percent of districts, with within-

[4] http://www.mext.go.jp/b_menu/toukei/chousa01/kihon/1267995.htm (Accessed Aug. 20, 2021).

district reporting discrepancies arising in only 13.8 percent of districts. This discrepancy prevalence is below the 21.6 percent among elementary students despite the smaller proportion of elementary students attending non-municipal schools (1.4 percent at the national level in 1994 (MEXT, 2018)), suggesting limited relevance of these students as sources of reporting discrepancies. Furthermore, in the vast majority of districts with mixed reports, the share of positive reports is two-thirds or more for both elementary and junior-high students, indicating that occasional nonattendance is the primary cause of reporting discrepancies. Additionally, only a small minority of districts have the share of positive reports between one-third and two-thirds, suggesting that most cases are clear-cut and misclassification due to data noise should be rare.

Comparing the derived school lunch status with official statistics further confirms the reliability of our method. As described in Appendix 1, municipal school lunch programs offer either "complete school lunch," "complementary school lunch," or "milk-only school lunch." According to the SLDB, 57.6-66.9 percent of Japanese municipal junior-high students had "complete school lunch," 0.5-0.7 percent had "complementary school lunch," and 18.9-26.5 percent had "milk only school lunch" during our study period. Different types of school lunch contents can obscure the causal interpretation of "having school lunch." We use "complete school lunch" and "complementary school lunch" as our definition of school lunch in our analysis, but exclude "milk-only school lunch" due to its limited scope. However, the NNS lack information on the type of school lunch, and it is unclear whether a respondent's report on school lunch in the NNS aligns with our conceptual notion of school lunch, warranting further investigation. To examine which type of school lunch is captured in our imputed school lunch status from the NNS, we conduct a prefecture-year level regression analysis, regressing the imputed school lunch status on the officially reported participation rates of the three types of school lunch. The dependent variable is the prefecture-year proportion of junior-high students reporting having school lunch in our sample. In constructing this variable, we relax the last two exclusion criteria in Figure A4, including districts with too few and/or conflicting reports

and districts where less than half of elementary students report having school lunch. We obtain data on the prefecture-year proportions of municipal junior-high students having "complete," "complementary," and "milk-only" school lunch from the SLDB. We then perform a regression analysis, regressing the variable constructed from our sample on the three SLDB variables.

The results are shown in Table A2. The coefficients on the proportions of students with "complete" and "complementary" school lunch are both significantly positive and close to one, implying that the vast majority of these students report having school lunch. The coefficient of the proportion of students with milk-only school lunch is not significant and has a small magnitude, implying that few of these students report having school lunch. These findings indicate that the school lunch information in the NNS data closely corresponds to the sum of "complete school lunch" and "complementary school lunch," which is our definition of school lunch.

Appendix 3. Details of Inverse Probability of Treatment Weighting (IPTW) and Propensity-Score Trimming

As a robustness check, we employ IPTW and propensity-score trimming in the DID analysis to balance municipal characteristics between the treatment and control groups in order to make the common trend assumption more plausible. We estimate the propensity scores for IPTW and propensity-score trimming using a logistic regression model in which the *NoSchoolLunch* dummy is regressed on district characteristics. Although the unit of observation in this regression is a child, all variation on the right-hand side is at the district level. The set of explanatory variables follows those in the district-level regression described in Subsection 4.4, but in accordance with Imbens (2015), we use numerical variables instead of dummy variables and omit insignificant variables to improve model fit. Specifically, we control for a linear time trend, prefectural population density, and the logged median population size for each municipal population size category obtained from the Statistics Bureau of Japan (2021a).

We then perform propensity-score trimming and exclude observations based on propensity scores to ensure sufficient overlap in characteristics between the treatment and control districts. We define the common support as the interval between the 1st and 99th percentiles of the estimated propensity scores, excluding from both the treatment and control groups observations outside of the common support (Stuart, 2010). We also exclude observations when the estimated propensity score is smaller than 0.1 or larger than 0.9 (Crump et al., 2009; Imbens, 2015). Finally, we assign an IPTW weight equal to the inverse of the probability of each subject's realized treatment status to each child in the trimmed sample. For students in district *d*, the weight equals $\frac{1}{\mathrm{p}_d}$ if d is a treatment district and $\frac{1}{1-\mathrm{p}_d}$ if d is a control district, where p_d denotes the propensity score of a lack of school lunch in district *d*. Under a set of assumptions, these weights allow for the consistent estimation of the average treatment effect (ATE), and they are referred to as ATE weights (Imbens 2004).

Table A3 compares normalized differences of individual and district characteristics between the treatment and control groups before and after IPTW and trimming for the full sample and the two low-SES subsamples. Normalized differences of the individual characteristics (i.e., the control variables in DID analysis) are shown in Panel (a) and those of district characteristics are shown in Panel (b). Both panels indicate that IPTW and trimming substantially reduce the absolute normalized differences. The absolute normalized difference occasionally increases slightly after these procedures, but only when the original value is small. When the original absolute normalized difference is large, these procedures always reduce it. While there is no agreed threshold for absolute normalized differences in the literature, Imbens (2015) suggests 0.30, the maximum absolute normalized difference in a randomized experiment, as a rule-of-thumb benchmark. In most of our analyses, the absolute normalized differences of individual characteristics are below 0.30, both with and without IPTW and trimming for all three samples. Some of the raw absolute normalized differences of district characteristics exceed 0.30 without IPTW and trimming, but most of them reduce to below 0.30 after IPTW and trimming. For a small number of samples used for robustness

checks, however, IPTW and trimming fail to reduce absolute normalized differences below 0.30. This typically occurs due to small sample sizes when we further divide low-SES subsamples by period and by urbanicity, and when we conduct subsample analysis of children with very low household expenditure and children with very high maternal BMI. The maximum of absolute normalized differences in DID-IPTW also exceeds 0.30 for the full sample when we relax the exclusion criterion of the prefectures and include all 47 prefectures, which might suggest large differences in district characteristics between the target and control groups in the added prefectures. Even in these cases, however, the absolute normalized differences are below 0.50.

The IPTW method can also recover the ATT using a different set of weights (ATT weights), where the weight of one is assigned to the treatment districts and the ratio of the propensity score of receiving treatment to that of receiving no treatment is assigned to the control districts. Thus, for students in district *d*, the weight equals 1 if *d* is a treatment district and $\frac{\mathrm{p}_d}{1-\mathrm{p}_d}$ if *d* is a control district, where p_d denotes the propensity score of a lack of school lunch in *d*. This scheme places larger weights on observations in the control group with a higher propensity score of being treated so that the control group resembles the treatment group. The results of IPTW-DID with ATT weights are shown in Table A4. The results are highly similar to those obtained using ATE weights (Panel (c) of Table 3), indicating no support for large effect heterogeneity between the treatment and control districts. This is consistent with the nonsignificant association of municipal provision of junior-high school lunch with resident characteristics and provides further support to our assumption that school lunch status is orthogonal to other determinants of body weight.

Appendix 4. Additional Analyses

Regression Analysis of NoSchoolLunch Dummy

We conduct a district-level Logit regression analysis in which we regress the *NoSchoolLunch* dummy on mean height, mean BMI, the prevalence rates of obesity and underweight among children aged 1 to 11, and other district characteristics. We use districts with five or more respondents aged 1 to 11. To minimize the influence of age composition among children, we use mean *z*-scores of heights and BMI that are normalized by gender, age, and five-year cohort, and employ the IOTF definitions for obesity and underweight. District characteristics are defined by aggregated NNS values: the number of NNS participants, age composition, median percentage ranking of per-member household expenditure, mean household size, occupational composition among 23- to 54-year-old workers, and the proportion of working women. Also included are prefectural population density obtained from the Statistics Bureau of Japan (2021a), dummies for municipal size, 47 prefecture-specific effects or six region-block-specific effects, and year dummies.

Table A5 shows the summary statistics of the district-level data. Junior-high lunch is less common in large municipalities. About 28 percent of the districts with junior-high lunch are in cities with a population of 150,000 or more, whereas about 55 percent of the districts without junior-high lunch are in cities with a population of 150,000 or more. Table A6 reports the regression results. Model 1 controls for region block fixed effects, and Model 2 controls for prefecture fixed effects. Junior-high lunch is less common in larger municipalities. The coefficient on the prefectural population density is significantly negative only in the prefecture fixed-effect model, implying a higher likelihood of school lunch provision in prefectures with a growing population. Consistent with this, school lunch is less common in areas with a larger fraction of the elderly population. Table A7 presents the estimated coefficients on year dummies of the *NoSchoolLunch* regression, which are not reported in Table A6. Consistent with the over-time increase in school lunch coverage (Figure A2), the estimated coefficients are significantly negative for most years since 1983 On the other hand, none of the other variables, including those directly related to obesogenic environments, are significant, as shown in Table A6.

Physical Fitness Test Score by Urbanicity and Survey Year

Our DID framework requires similar age trends in other determinants of body weight, apart from school lunch, between the treatment and control groups. Lower junior-high school lunch coverage in more urban areas raises concern that age trends in the physical activity level might vary by urbanicity. Junior-high students typically spend more time studying and on school sports club activities than elementary students (Cabinet Office, 2015), and urban junior-high students might spend more time studying and less time on sports than their rural counterparts. Because the NNS contains limited reliable information on children's physical activity levels, we use the physical fitness test score as a proxy for the physical activity level and compare age trends of the score by urbanicity.

We use aggregate data on municipal elementary and junior-high school students in grades 5-9 (ages 10-15) retrieved from the Physical Fitness and Athletic Performance Survey conducted by the Ministry of Education. The physical fitness test consists of seven items (side-stepping, vertical jump, back strength, hand-grip strength, backward-bending of the upper body, anteflexion in standing, and modified Harvard step test). Each item is evaluated on a scale of 1-10 and the test score is the sum of the item scores. Students are grouped into three urbanicity categories: rural areas (defined as sparsely populated countryside rich in natural environment); urban areas (defined as urbanized areas with a fair amount of natural environment, playgrounds, and sports grounds); and congested areas (defined as urban areas where residences, enterprises, and shops concentrate, with limited natural environment, playgrounds, and sports grounds).

Panel (a) of Figure A5 plots children's mean physical fitness test score in 1984, the mid-year of our study period, over school grade by urbanicity category, separately for boys and girls. Children in school grades 5 and 6 are elementary students (ages 10-12), and those in grades 7-9 are junior-high

students (ages 12-15); vertical red lines indicate a school grade of 6.5, the threshold that divides elementary and junior-high students. For both genders and across school grades, the score is negatively associated with urbanicity, but there is no clear indication that the magnitude of the association differs between junior-high and elementary students. These findings do not support significant differences in age trends in the physical activity level by urbanicity.

Similarly, the increase in junior-high lunch coverage during our study period raises concern that age trends in the physical activity level might have changed over time. Panel (b) of Figure A5 plots the mean test scores for elementary students in grade 5 (ages 10-11) and junior-high students in grade 7 (ages 13-14) over the period between 1975 and 1994, separately for boys and girls. There are no consistent chronological trends in either the score by grade or the difference between the grades, which does not support significant over-time changes in age trends in the physical activity level.

Changing Cut-Off Values for Low Household Expenditure

Dividing the sample using different cut-off values of per-member household expenditure may enable exploration of effect heterogeneity by household expenditure. However, data limitations hinder conducting detailed analysis in this regard. First, per-member household expenditure is reported by category, which varies by year, and the categorization is too crude to finely segment the sample by percentile ranges. There are only eight categories, and children are unevenly distributed across these categories in a time-varying manner. For instance, in 1979, approximately 44 percent of children are in the highest category, while in 1980, approximately 55 percent are in the lowest three categories. The second data limitation is the sample size in each district. The DID analysis requires at least one elementary student and one junior-high student in each district, and in our subsample of children with below-median household expenditure, the median number of children in a district is seven, with many districts barely meeting this requirement. Thus, restricting the sample to a very low percentile

of per-member household expenditure excludes many districts which have an insufficient number of children below the threshold.

Nevertheless, we conduct additional analysis by restricting the sample to children with low household expenditure, using the 40th, 60th, and 70th percentile thresholds in addition to the median threshold in the main analysis. The results are shown in Table A8. Overall, the estimated effects are larger for lower percentiles, suggesting larger weight reduction effects of school lunch for children with lower household expenditure. We do not use smaller thresholds than the 40th percentile because the number of districts in the sample of children with below the 40th percentile per-member household expenditure is 861 after propensity score trimming, with only 177 districts in the treatment group. This suggests that using a lower threshold further reduces the sample size and would not allow us to estimate the effect precisely.

Cost-Effectiveness Analysis

We conduct a back-of-the-envelope calculation to examine the cost-effectiveness implications of our main analysis results. We refrain from cost-benefit analysis due to the scarcity of monetary evaluations of benefits terms from reducing child obesity in Japan, which stems from the lack of prior evidence. While a recent study estimates the causal effect of child obesity on healthcare costs in the US (Biener et al., 2020), we know of no study exploring the monetary cost of child obesity in Japan. Japanese medical studies have discovered significantly large medical care costs for obese adults (Nagai et al., 2012) and a strong persistence of obesity from childhood to adulthood (Togashi et al., 2002); however, these studies utilize datasets with limited representativeness and provide non-causal estimates. We also confine our analysis to the benefits of obesity reduction and disregard various other school lunch benefits. A comprehensive cost-benefit analysis is challenging because school lunch may have a variety of difficult-to-measure benefits, such as various health

improvements (Gundersen et al., 2012; Lundborg et al., 2021), improved academic performance (Belot and James, 2011; Anderson et al., 2018), and increased maternal labor force participation (Lundborg et al., 2021).

We calculate the public cost per obesity case prevented by dividing the mean public expenditure on school meals per child by the estimated obesity reduction effect of school lunch for a child. As we find a statistically significant obesity reduction effect of school lunch only for low-SES subsamples, we evaluate the obesity reduction effect for the full sample by multiplying the obesity reduction effect for a subsample by the subsample's share of the number of children. We focus on obesity prevalence under the IOTF definition because the non-negligible prevalence of underweight individuals in Japan makes health benefits from weight reduction difficult to interpret, and the IOTF definition is more internationally comparable than the POW definition. Estimates for obesity status under the POW definition closely resemble those under the IOTF definition.

We calculate the public expenditure on school meals per child as follows. Following Nomura (2020), we define public expenditure on school meals as municipal expenditure on labor, property, and maintenance and repair costs for school meals. Data on municipal expenditure by fiscal year are retrieved from the Local Finance Statistics Annual Report conducted by the Ministry of Internal Affairs and Communications. Data on the annual number of students with school lunch (as of May 1st) are retrieved from the School Lunch Data Book (SLDB). The number of children receiving publicly funded school lunch is determined by adding the number of municipal-school students who have "complete" or "complementary" school lunch. We obtain the mean annual public expenditure on school meals per child by dividing the mean annual public expenditure by the mean annual number of children with school lunch and converting the value to fiscal year 2020 values using the Consumer Price Index (CPI), excluding imputed rent (Statistics Bureau of Japan, 2021b). Since data on the number of students with school lunch are missing for 6 out of 20 years, we estimate a linear regression model of the annual public expenditure per child on the year and use the fitted value at the

mean year of 1984.5 as the mean value over the 20 years. Finally, we convert this value to USD in fiscal year 2020 values.

Our estimate of the mean annual per-capita public expenditure on school lunch is approximately 40 thousand yen (377 USD) in 2020 values. Based on the base DID estimate for children with non-white-collar fathers, the estimated cost per obesity case prevented is 2,347 thousand yen (22,133 USD), and based on that for children with low household expenditure, it is 1,895 thousand yen (17,870 USD). While it is difficult to compare these estimates with previous estimates on other interventions due to differences in obesity definitions and health risks associated with excessive weight, the Japanese school lunch program appears cost-effective when compared to other interventions estimated to significantly reduce child obesity. For instance, in the US, the national dissemination of the Nutrition and Physical Activity Self-Assessment for Child Care (NAP SACC) program from 2015 to 2025 has an estimated cost per obesity case prevented of 19,044 USD, where obesity is defined as a BMI at or above the 95th percentile for children of the same age and sex (Gortmaker et al., 2015). This definition resembles our IOTF obesity definition in that it defines obesity by the BMI percentile by age and sex and results in an obesity prevalence of approximately five percent, similar to the obesity rate under the IOTF definition among junior-high students of about six percent in our sample.

Appendix 5. Details of Robustness Checks

Permutation Test

To address concerns in our DID analysis that correlation among children close in age and location might lead to underestimation of standard errors, we implement non-parametric permutation tests following the approaches of Bertrand et al. (2004) and Abadie et al. (2010). In this test, the treatment *NoSchoolLunch* is randomly assigned to control districts, maintaining the same frequency as in the

original sample, and the base DID model is estimated based on the hypothetical treatment assignment. Repeating this procedure many times yields the distribution of the estimated placebo treatment effect. The resulting placebo distributions from 1,000 random draws are shown in Figure A6 for (a) the full sample, (b) the subsample of children with non-white-collar fathers, and (c) the subsample of children with low household expenditure in Panels (a) to (c), respectively. The vertical red lines indicate the actual estimate, i.e., the estimated treatment effect in the original estimation. Numbers below each graph show the actual estimate and the pseudo *P*-value, defined as the frequency ratio of placebo estimates exceeding the actual estimate in absolute terms. The results are highly consistent with our main findings from the base DID presented in panel (b) of Table 3. For BMI, BMI *z*-score, POW, and the two obesity measures, the actual estimates in all three samples consistently appear in the right tail of the distribution, and in both low-SES subsamples, the pseudo *P*-values are all smaller than 0.05, while the pseudo *P*-values in the full sample are all larger than 0.1. For underweight status, the actual estimates are around the middle of the distribution, and the pseudo *P*-values are larger than 0.1 in all three samples. These findings imply that the statistical significance of our estimated school lunch effects is not due to misspecification of the correlation structure.

Falsification Test: Regression Analysis of Height

We conduct a falsification test, using height instead of weight measures as the regressand. Because height is determined primarily by genetic factors and early-life environment, and the effect of adolescent lifestyle is limited (Beard and Blaser, 2002), school lunch should not have a strong, immediate effect on height. This is not the case if children suffer from serious malnutrition, especially protein deficiency (Grasgruber et al., 2014), but the prevalence of protein deficiency in our sample is only 0.55 percent (Appendix 2). We test the null hypothesis of identical differences in

height between elementary and junior-high students in treatment and control groups using the coefficient of the interaction of the *JuniorHigh* dummy and the *NoSchoolLunch* dummy in the regression of height. As shown in Table A9, we find that none of the DID estimates are significant for both the full sample and the low-SES subsamples, providing additional support for the common trend assumption.

Event study analysis

To further examine if our results are driven by differential growth patterns between the treatment and control groups that emerge before children enter junior high, we add interactions of the *NoSchoolLunch* dummy with age dummies to the DID regression model and explore how the DID estimate evolves with age. This is not an event study model in a strict sense, as the DID model uses age instead of time. Additionally, the age-specific DID estimates for junior high students do not precisely capture the dynamic effect of the treatment, because each age group (by one year) contains children in two school grades: For instance, 13-year-old junior high students consist of seventh and eighth graders. Unfortunately, we cannot estimate grade-specific DID estimators due to data limitations, as detailed in Appendix 2. Nevertheless, the age-specific DID estimates for elementary students would capture pre-existing differential trends in growth patterns, if they exist.

The results are presented in Figure A7. 12-year-olds are excluded from this analysis because our sample excludes them for 1975–1985 due to data limitations. 14 and 15 years are categorized into the same age group due to the small number of 15-year-old junior high students. The DID estimate for the reference age group of 11 years is normalized to zero. Triangles and circles show the DID estimates for elementary students (age 9, 10, 11) and junior high students (age 13, 14-15), respectively, and the line segments show the 95 percent confidence intervals. None of the DID estimates for elementary students are significant or show consistent increasing or decreasing patterns, which provides no evidence of significant pre-existing differential trends. In contrast, the DID

estimates for junior high students are all positive and most of them are statistically significant, except for those for obesity measures in children with non-white-collar fathers. Overall, they are close in magnitude for both age groups.

Appendix References

Abadie A, Diamond A, Hainmueller J, 2010. Synthetic control methods for comparative case studies: estimating the effect of California’s tobacco control program. *Journal of the American Statistical Association*, 105:493–505.

Anderson ML, Gallagher J, Ritchie ER, 2018. School lunch quality and academic performance. *Journal of Public Economics*, 168:81–93.

Asahi Shimbun, 1993, October 27. *The Optional School lunch programs at junior high Schools in Nagoya Had a 60% Utilization Rate, Falling below Expectations*. Retrieved from Asahi Shimbun Kikuzo database (In Japanese).

Asahi Shimbun, 1996, August 27. *Shaken by O157: Turning Point for Uniformly Imposed School Lunch*. Retrieved from Asahi Shimbun Kikuzo database (In Japanese).

Beard AS, Blaser MJ, 2002. The ecology of height: the effect of microbial transmission on human height. *Perspectives in Biology and Medicine*, 45(4):475–498.

Belot M, James J, 2011. Healthy school meals and educational outcomes. *Journal of Health Economics*, 30(3):489–504.

Bertrand M, Duflo E, Mullainathan S, 2004. How much should we trust differences-in-differences estimates? *Quarterly Journal of Economics*, 119(1):249–275.

Biener AI, Cawley J, Meyerhoefer C, 2020. The medical care costs of obesity and severe obesity in youth: an instrumental variables approach. *Health Economics*, 29(5):624–639.

Cabinet Office, 2015. *White Paper on Youth 2007 in Japan* (In Japanese). https://www8.cao.go.jp/youth/whitepaper/h27honpen/index.html (Accessed August 10, 2022).

City of Sapporo, 1997. *Proposal on the Future State of School Lunch in the City of Sapporo* (In Japanese). https://www.city.sapporo.jp/kyoiku/top/kyushoku/plan/teigen.html (Accessed March 10, 2021).

Crump R, Hotz JV, Imbens G, Mitnik O, 2009. Dealing with limited overlap in estimation of average treatment effects. *Biometrika*, 96(1):187–199.

Gortmaker SL, Wang YC, Long MW, Giles CM, Ward ZJ, et al., 2015. Three interventions that reduce childhood obesity are projected to save more than they cost to implement. *Health Affairs*, 34(11):1932–1939.

Grasgruber P, Cacek J, Kalina T, Sebera M, 2014. The role of nutrition and genetics as key determinants of the positive height trend. *Economics & Human Biology*, 15:81–100.

Gundersen C, Kreider B, Pepper J, 2012. The impact of the National School Lunch Program on child health: a nonparametric bounds analysis. *Journal of Econometrics*, 166:79–91.

Hironaka Y, Kasai E, 2019. Current situation and problems of school lunch: cost performance of school lunch. *Production Management: Journal of Japan Society for Production Management*, 26(2):71–76 (In Japanese).

Hosoya N, Fukui T, Kobayashi S, Hashimoto I, Innan S, et al., 1985. On the newly developed nutritional allowance guidelines. *Journal of Japan Society of Nutrition and Food Sciences*, 38(3):201–215 (In Japanese).

Imbens GW, 2004. Nonparametric estimation of average treatment effects under exogeneity: a review. *Review of Economics and Statistics*, 86(1):4–29.

Imbens GW, 2015. Matching methods in practice: three examples. *Journal of Human Resources*, 50(2):373–419.

Kawamura I, 1995. Surgical treatment of obesity. *Journal of the Japanese Society of Internal Medicine*, 84(8):1295–1299 (In Japanese).

Lundborg P, Rooth DO, Alex-Petersen J, 2021. Long-term effects of childhood nutrition: evidence from a school lunch reform. *Review of Economic Studies*, 89(2):876–908.

Mainichi, 2015, December 10. *Himeji City Board of Education to Warn 18 Children Suspected for Attending a School out of School District within This Year*. Retrieved from mainichi.jp (In Japanese).

Maruyama S, Nakamura S, 2018. Why are women slimmer than men in developed countries? *Economics & Human Biology*, 30:1–13.

MEXT, 2018. *Statistical Abstract of Education* (In Japanese). https://www.mext.go.jp/b_menu/toukei/002/002b/1403130.htm (Accessed Oct. 5, 2021).

MHLW 2018, *Overview of Comprehensive Survey of Living Conditions 2018* (In Japanese). https://www.mhlw.go.jp/toukei/saikin/hw/k-tyosa/k-tyosa18/index.html (Accessed Aug. 20, 2021).

Nagai M, Kuriyama S, Kakizaki M, Ohmori-Matsuda K, Sone T, Hozawa A, Kawado M, Hashimoto S, Tsuji I, 2012. Impact of obesity, overweight and underweight on life expectancy and lifetime medical expenditures: the Ohsaki Cohort Study. *BMJ Open*, 2:e000940.

Nagoya Narumi Junior High School, 2019. *School History* (In Japanese). https://www.nagoya-c.ed.jp/school/narumi-j/service.html (Accessed March 10, 2021).

Nakamura M, 2000. School choice in Japan: learning from the experiences of other countries. *Nagano Technical College Research Reports*, 34:107–116.

Narusaka M, 1996. Changes in fat supply in school lunch menus in Okayama City. *Japanese Journal of Nutrition and Dietetics*, 54(2):121–128 (In Japanese).

National Federation of University Co-operative Associations, 2020. *Campus Life Data 2020.* Tokyo: National Federation of University Co-operative Associations. (In Japanese).

Nichirei Foods, 2019. *Survey on Packed Lunches* (In Japanese). https://www.nichireifoods.co.jp/research/obento2019/ (Accessed March 4, 2021).

Nomura K, 2020. Development of education services production database of Japan (ESJ), 1955–2017. *New ESRI Working Paper* No. 51 (In Japanese). https://www.esri.cao.go.jp/jp/esri/archive/new_wp/new_wp050/new_wp049.pdf (Accessed Aug. 20, 2021).

Norinchukin Bank, 2017. *The Third Survey of Diet, Awareness, and the Actual Situation of Contemporary High School Students* (In Japanese). https://www.nochubank.or.jp/efforts/research.html (Accessed August 19, 2021).

Nozue M, 2011. The contribution of school lunch to dietary intakes in school children and application of dietary reference intakes: a case study of fifth-grade school children. *Ph.D. Thesis, Kagawa Nutrition University* (In Japanese).

Sasaki S, 2018. Determinant factors for time spent on housework by men at the era of work-life balance in Japan. *Journal of Household Economics*, 47:47–66 (In Japanese).

Statistics Bureau of Japan, 2021a. *Historical Statistics of Japan*. https://www.stat.go.jp/english/data/handbook/index.html (Accessed Aug. 20, 2021).

Statistics Bureau of Japan, 2021b. *Report on the Consumer Price Index: Historical Data*. https://www.stat.go.jp/english/data/cpi/1588.html#his (Accessed Aug. 17, 2021).

Stuart EA, 2010. Matching methods for causal inference: a review and a look forward. *Statistical Science*, 25(1):1–21.

Suzuki, MH, 2016. Endocrine and metabolic emergencies: points of initial management. *Journal of the Japanese Society of Internal Medicine*, 105(4):676–682 (In Japanese).

Togashi K, Masuda H, Rankinen T, 2002. A 12-year follow-up study of treated obese children in Japan. *International Journal of Obesity*, 26:770–777.

Figure A1. School lunch scenes in Japan

a) A school lunch line

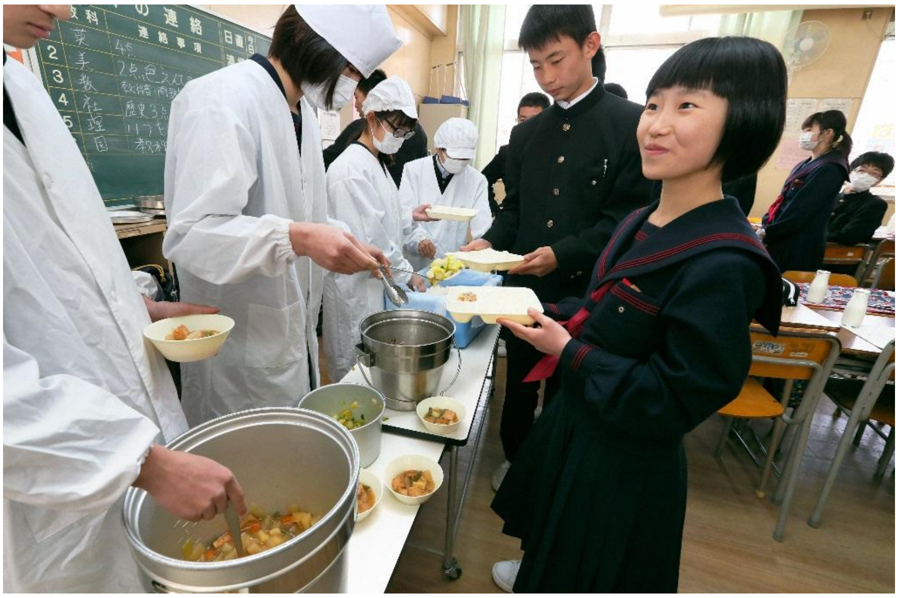

Source: “School Lunch at Oyodo Junior High, Osaka City,” March 8, 2017, retrieved from Asahi Shimbun Photo Archive.

b) Students eating together

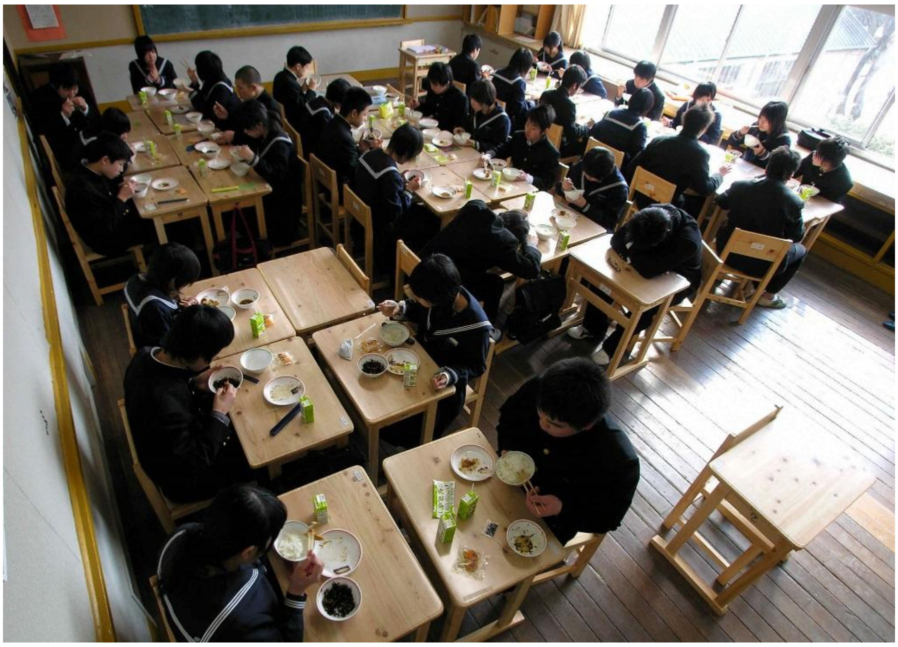

Source: “School Lunch at Aoyama Junior High, Beppu City,” January 24, 2007, retrieved from Asahi Shimbun Photo Archive.

Figure A2. Trends in the school lunch coverage rate for municipal junior-high students

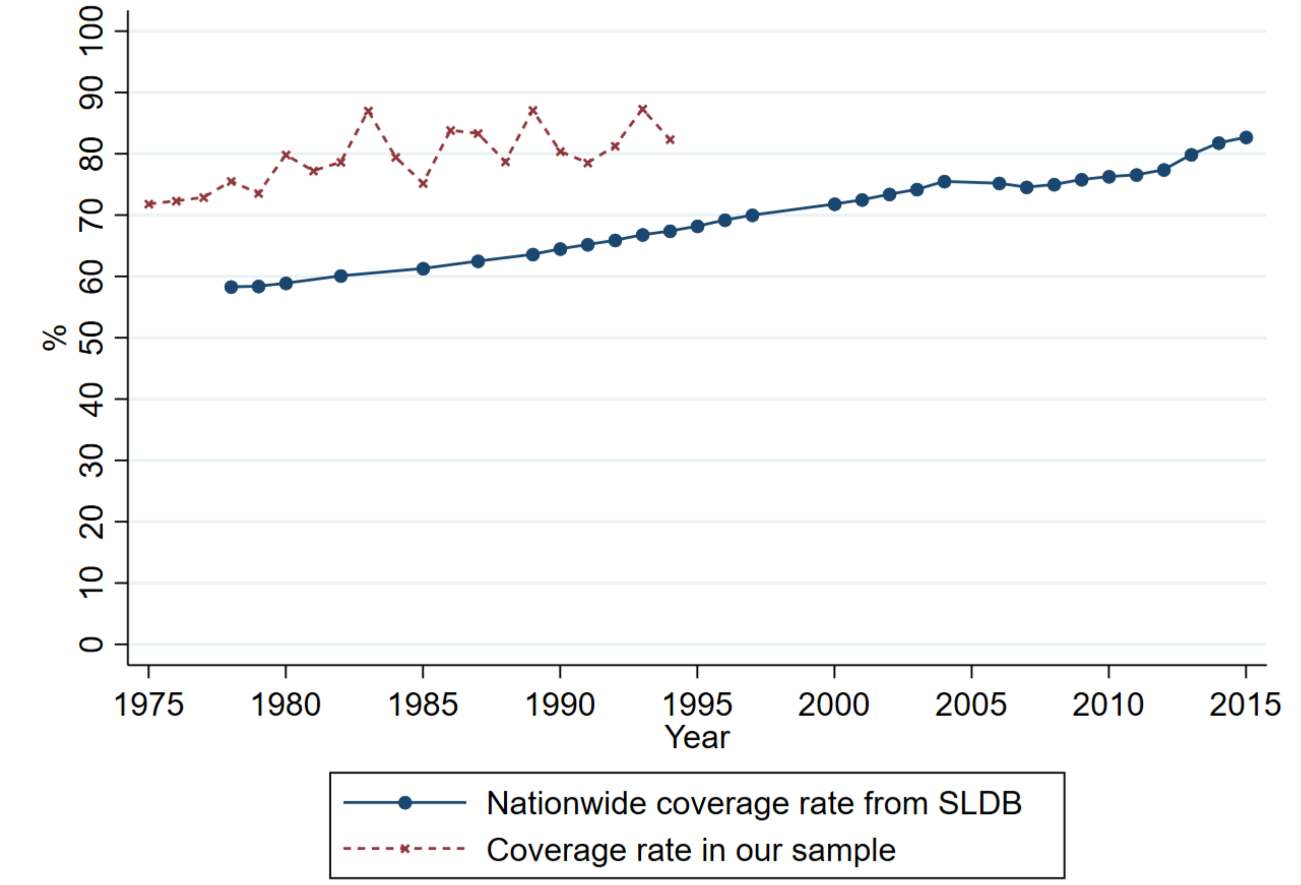

Note: The nationwide coverage rate is based on the School Lunch Data Book (SLDB) and defined as the share of municipal junior-high students who have school lunch. The sample coverage rate is the share of junior-high students in districts with school lunch in our sample. For the discussion on the difference between the two coverage rates, see Appendix 1.

Figure A3. Relationship among the school type, grade, and age

<table>
<tr><td>Sample</td><td colspan="13">9- to 15-year-olds in compulsory education</td></tr>
<tr><td>School type (unobservable)</td><td colspan="7">Elementary</td><td colspan="6">Junior High</td></tr>
<tr><td>School grade (unobservable)</td><td>3</td><td colspan="2">4</td><td colspan="2">5</td><td colspan="2">6</td><td colspan="2">7</td><td colspan="2">8</td><td colspan="2">9</td></tr>
<tr><td>Age (observable)</td><td colspan="2">9</td><td colspan="2">10</td><td colspan="2">11</td><td colspan="2">12</td><td colspan="2">13</td><td colspan="2">14</td><td>15</td></tr>
</table>

Note: Children’s school grade in Japan is strictly determined by the child’s age on April 2nd. The NNS questionnaire asks if children are in compulsory education but does not distinguish between elementary schools and junior highs. We use the children’s age to categorize 9- to 11-year-olds and 13- to 15-year-olds as elementary and junior-high students, respectively. In the 1975–1985 NNS, the school grade of 12-year-olds cannot be determined, but for those who are 12 years old in 1986–1994, we can use the birth month information. We exclude 12-year-olds for 1975–1985 and November-born 12-year-olds for 1986-1994 from the sample because we cannot determine their school grade.

Figure A4. Sample exclusion criteria and changes in the sample size

Step	Sample
Initial sample: 9- to 15-year-old children in compulsory education, excluding 12-year-olds in the 1975–1985 NNS and November-born 12-year-olds in the 1986–1994 NNS	5,587 districts, 38,225 children
Exclude children in prefectures with a high proportion of junior-high students in non-municipal schools and children in the city of Nagoya in 1993 and 1994	↓
	3,280 districts, 24,614 children
Exclude children without valid information on height and weight	↓
	3,228 districts, 22,241 children
Exclude children with deviant height and weight	↓
	3,227 districts, 22,205 children
Exclude children without a mother in the household or without valid household expenditure data	↓
	3,205 districts, 21,328 children
Exclude districts without elementary students and districts without junior-high students	↓
	2,661 districts, 20,065 children
Exclude districts with conflicting or too few answers on school lunch	↓
	2,317 districts, 18,703 children
Exclude districts without school lunch for elementary school children	↓
	2,271 districts, 18,305 children

Figure A5. The physical fitness test score

Panel (a): The mean test score over school grade in 1984

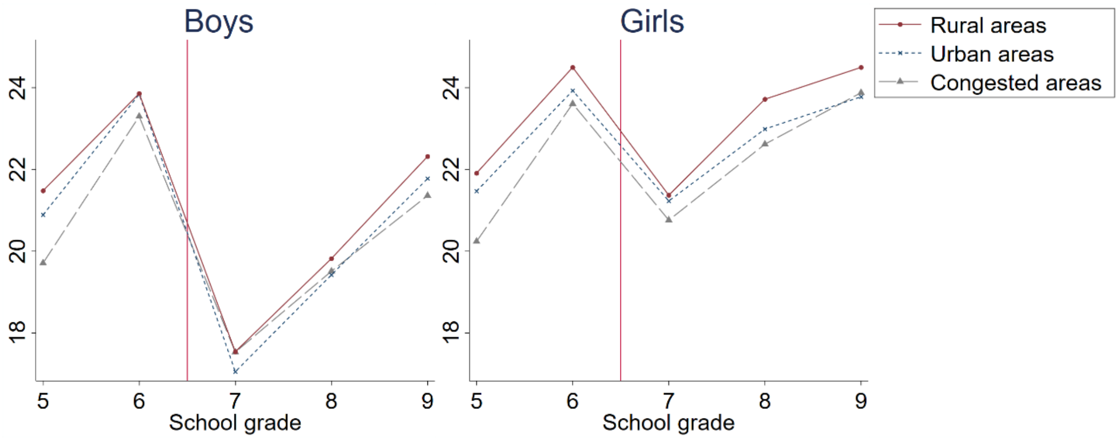

Panel (b): Trends in the mean test score

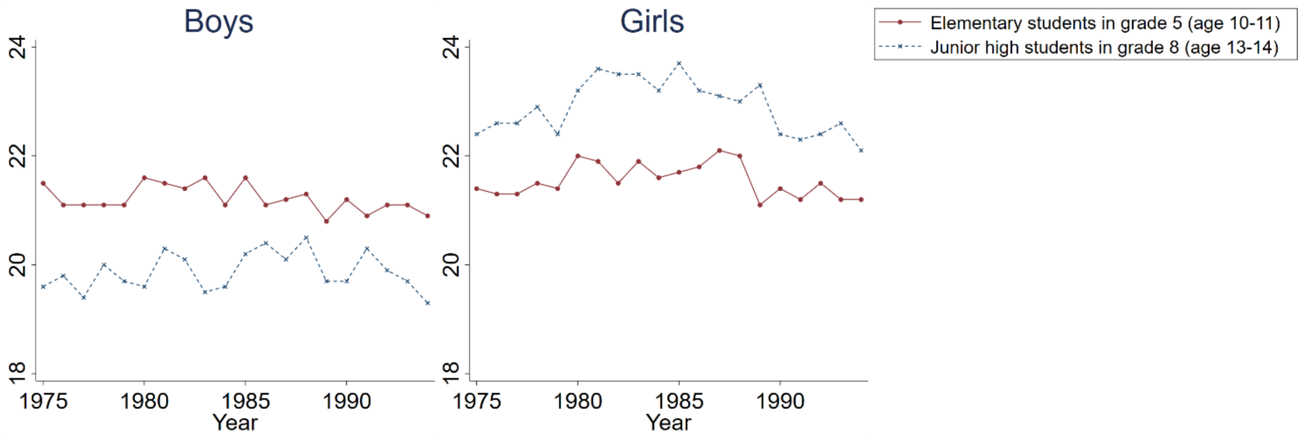

Note: We use aggregate data on elementary and junior high students attending municipal schools from the Physical Fitness and Athletic Performance Survey conducted by the Ministry of Education. The physical fitness test consists of seven items (side stepping, vertical jump, back strength, hand-grip strength, backward-bending of the upper body, anteflexion in standing, and modified Harvard step test). Each item is evaluated on the scale of 1-10 and the test score is the sum of the item scores. In panel (a), vertical red lines indicate school grade of 6.5, the threshold that divides elementary and junior-high students.

Figure A6. Permutation tests: empirical distributions of placebo estimates

Panel (a): Full sample

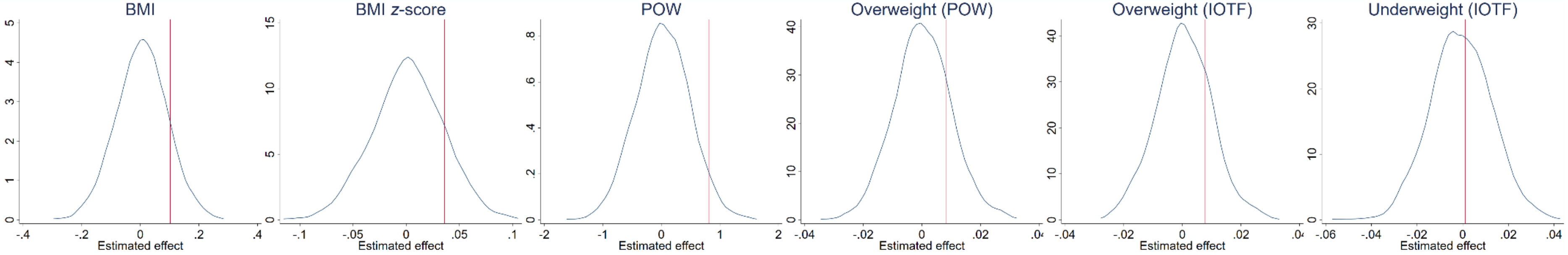

p=0.238 *p*=0.283 *p*=0.079 *p*=0.389 *p*=0.408 *p*=0.939

Panel (b): Children with non-white-collar fathers

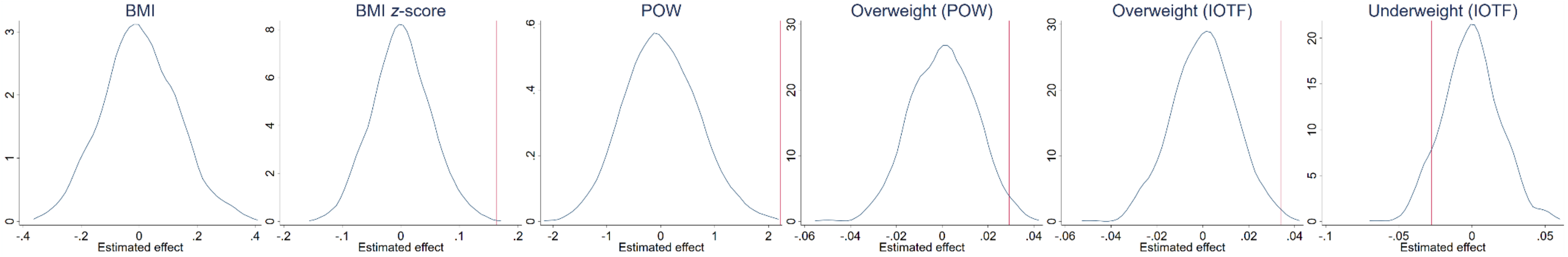

p=0 *p*=0 *p*=0 *p*=0.032 *p*=0.008 *p*=0.163

Note: Each graph shows the kernel density estimate of the distribution of estimated placebo treatment effects using the Epanechnikov kernel. The vertical red line represents the actual estimate. "*p*" refers to the pseudo *P*-value defined as the frequency ratio of placebo estimates exceeding the actual estimate in absolute terms (in 1,000 random draws).

Figure A6 (cont.)

Panel (c): Children with low household expenditure

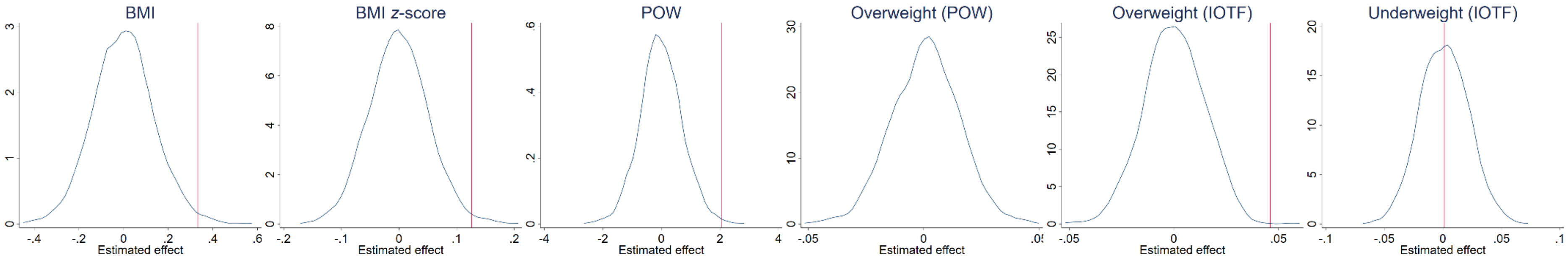

p=0.018 *p*=0.016 *p*=0.009 *p*=0 *p*=0.003 *p*=0.956

Note: Each graph shows the kernel density estimate of the distribution of estimated placebo treatment effects using the Epanechnikov kernel. The vertical red line represents the actual estimate. "*p*" refers to the pseudo *P*-value defined as the frequency ratio of placebo estimates exceeding the actual estimate in absolute terms (in 1,000 random draws)

Figure A7: DID estimates by age group

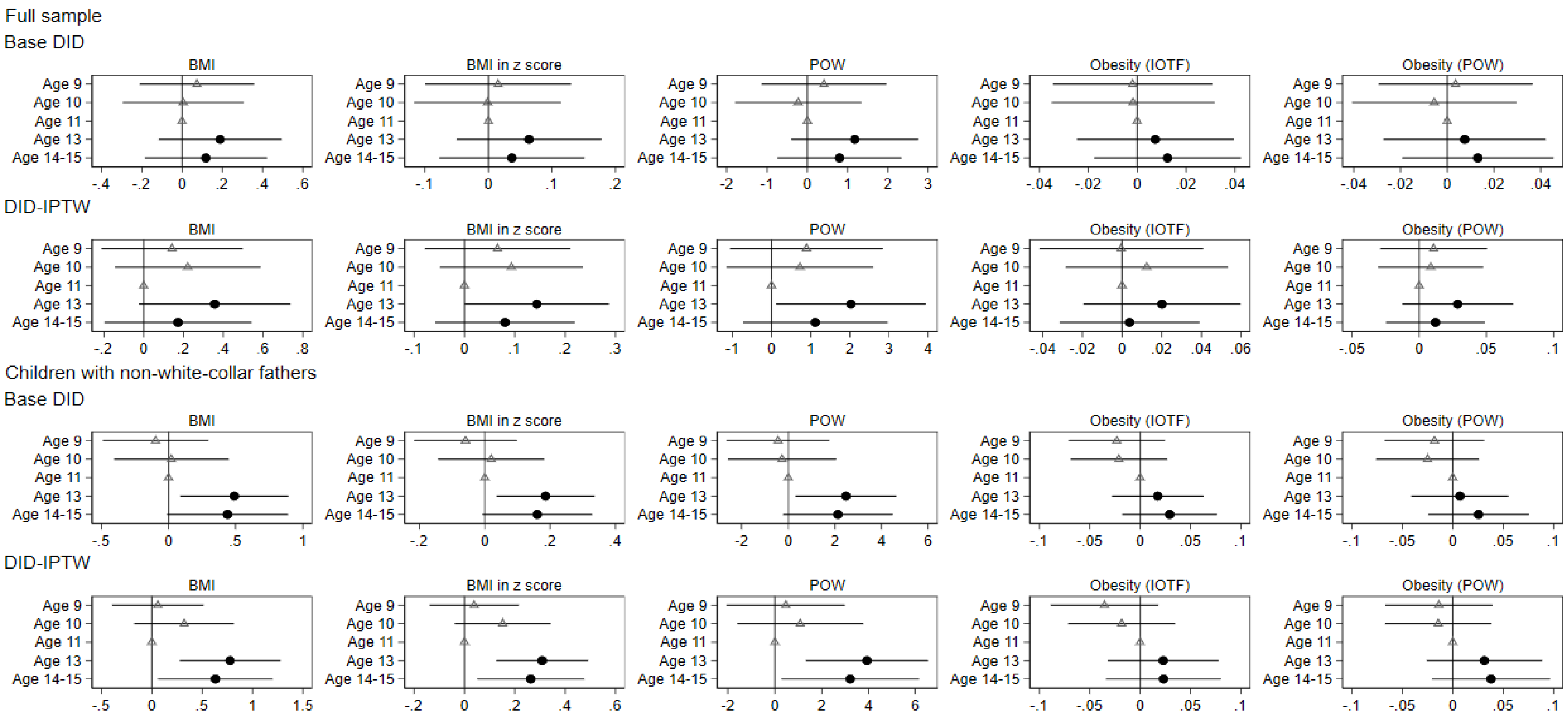

Note: 12-year-olds are excluded from the analysis because our sample excludes them for 1975–1985 due to data limitations. The reference age group is 11 years. 14 and 15 years are categorized into the same age group due to the small number of 15-year-old junior high students. Triangles and circles show the DID estimates for elementary students and junior high students, respectively, and the line segments show the 95 percent confidence intervals.

Figure A7 (cont.)

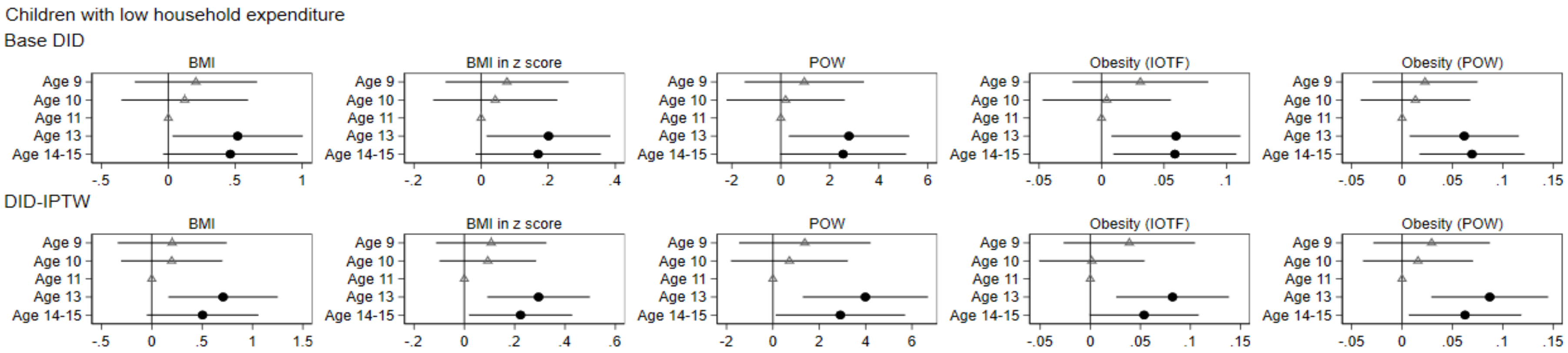

Note: 12-year-olds are excluded from the analysis because our sample excludes them for 1975–1985 due to data limitations. The reference age group is 11 years. 14 and 15 years are categorized into the same age group due to the small number of 15-year-old junior high students. Triangles and circles show the DID estimates for elementary students and junior high students, respectively, and the line segments show the 95 percent confidence intervals.

Table A1. The proportion of students reporting school lunch by district

Share of students who reported having school lunch at least once during the survey period	Elementary students		Junior-high students	
No student	36	(1.6%)	470	(20.6%)
more than 0 and less than 1/3	18	(0.8%)	36	(1.6%)
1/3 or more and less than 1/2	5	(0.2%)	24	(1.1%)
1/2 or more and less than 2/3	17	(0.7%)	44	(1.9%)
2/3 or more and less than 1	451	(19.8%)	210	(9.2%)
All students	1,751	(76.9%)	1,494	(65.6%)
Total number of districts	2,278	(100.0%)	2,278	(100.0%)

Note: The unit of observation is a district. The figures are based on the sample before we exclude districts with exactly two conflicting reports and districts where 50% or less of elementary students had school lunch (see Appendix 2). Districts with one or no reports are not included.

Table A2. Prefecture-year level regression of the proportion of junior-high students who had school lunch

Variable	Coefficients	
SLDB proportion of students with complete school lunch	0.893***	(0.092)
SLDB proportion of students with complementary school lunch	0.947**	(0.458)
SLDB proportion of students with milk only	0.031	(0.107)
Constant	8.083	(8.863)
N	427	
Adjusted R^2	0.320	

Note: Standard errors are in parentheses. The unit of observation is a prefecture-year. The proportion of junior-high students who had school lunch in our NNS sample is calculated at the prefecture-year level and regressed on the proportions of those with the three types of school lunch in the official SLDB statistics. Both the regressand and the regressors are in percentage terms. *, **, and *** indicate statistical significance at the 10%, 5%, and 1% levels, respectively.

Table A3. Normalized differences of individual and district characteristics

Panel (a): Individual characteristics (control variables in DID analysis)

Variable	Full sample		Children with non-white-collar fathers		Children with low household expenditure	
Trimming and IPTW-weighting	No	Yes	No	Yes	No	Yes
Male	0.013	0.008	0.023	0.046	0.011	-0.005
Age	-0.024	-0.020	-0.043	-0.039	0.004	0.020
Father's age	0.005	-0.022	-0.001	-0.051	0.010	0.023
Father's height (*z* score by age, sex, and 5-year cohort)	0.072	0.026	0.042	-0.002	0.045	0.034
Father's BMI (*z* score by age, sex, and 5-year cohort)	-0.035	0.000	-0.033	-0.038	-0.036	-0.032
Father's height and BMI missing	0.010	-0.004	-0.041	-0.080	-0.002	-0.040
Father: white-collar worker (the reference category)	0.029	-0.001			0.004	-0.022
Father: laborer	0.009	-0.001	0.061	0.020	0.031	-0.004
Father: self-employed	0.016	-0.023	0.027	-0.064	0.025	0.008
Father: agriculture/fisheries/forestry	-0.102	0.021	-0.128	0.068	-0.088	0.034
Father: other occupation (not working)	-0.003	-0.025	-0.008	-0.025	0.025	-0.001
Without father in household	0.003	0.023			-0.005	-0.010
Mother's age	0.000	0.016	-0.048	-0.049	-0.017	0.011
Mother's height (*z* score by age, sex, and 5-year cohort)	0.056	-0.008	0.008	-0.034	0.017	-0.034
Mother's BMI (*z* score by age, sex, and 5-year cohort)	-0.097	-0.053	-0.080	-0.059	-0.096	-0.054
Mother's height and BMI missing	0.000	0.022	0.002	0.041	-0.045	-0.036
Mother: white-collar worker (the reference category)	0.023	0.070	0.057	0.072	0.044	0.060
Mother: laborer	-0.022	0.040	-0.020	0.055	0.005	0.040
Mother: self-employed	0.023	0.010	0.024	-0.004	0.056	0.068
Mother: agriculture/fisheries/forestry	-0.150	-0.039	-0.163	-0.017	-0.168	-0.072
Mother: other occupation (not working)	0.066	-0.074	0.076	-0.084	0.041	-0.085
Grandfather in household	-0.100	0.019	-0.124	-0.002	-0.076	0.030
Grandmother in household	-0.096	0.037	-0.073	0.038	-0.043	0.083
# of children in household (below 18 years old)	-0.042	0.002	-0.029	-0.032	-0.012	-0.003
Per-member household expenditure (defined as 100% = families with lowest expenditures in each survey year)	-0.149	0.034	-0.179	-0.043	-0.177	-0.130

Note: None of the absolute normalized differences exceed 0.30.

Table A3. (cont.)

Panel (b): District characteristics

Variable	Full sample		Children with non-white-collar fathers		Children with low household expenditure	
Trimming and IPTW-weighting	No	Yes	No	Yes	No	Yes
Year	-0.222	0.061	-0.247	0.003	-0.131	0.096
Prefectural population density (1,000 person/km2)	0.063	-0.128	0.084	-0.078	0.025	-0.123
Logged municipal population size	0.726*	-0.056	0.799*	-0.016	0.677*	-0.047
District: mean child height (*z* score)	0.126	0.117	0.12	0.079	0.176	0.116
District: mean child BMI (*z* score)	-0.035	0.049	-0.033	0.003	0.018	0.088
District: child obesity rate (IOTF)	0.026	0.08	0.064	0.08	0.02	0.071
District: child underweight rate (IOTF)	0.046	-0.035	0.057	-0.026	-0.018	-0.064
District: # of participants	-0.145	-0.075	-0.103	-0.09	-0.09	-0.035
District: mean age	-0.076	0.17	-0.095	0.124	-0.046	0.230
District: median per-member household expenditure	-0.329*	-0.045	-0.348*	-0.098	-0.316*	-0.092
District: mean household size	-0.21	-0.012	-0.201	-0.019	-0.177	0.000
District: proportion of white-collar worker	0.062	-0.044	0.01	-0.125	0.051	-0.102
District: proportion of laborer	0.022	0.034	0.069	0.066	0.067	0.049
District: proportion of self-employed	0.126	0.025	0.171	0.025	0.116	0.034
District: proportion of agriculture	-0.222	-0.004	-0.219	0.025	-0.194	0.023
District: proportion of working women	-0.138	0.193	-0.128	0.203	-0.122	0.197

Note: * indicates absolute normalized difference exceeding 0.30. Occupational proportions refer to the proportion of 23- to 54-year-old workers in each occupation.

Table A4. Effect of no school lunch: DID-IPTW using ATT weights

Sample	# children	# districts	BMI	BMI z-score	POW	Obesity (IOTF)	Obesity (POW)	Underweight (IOTF)
Full sample	13,985	1,741	0.092	0.042	0.829	0.003	0.008	0.003
			(0.107)	(0.041)	(0.545)	(0.011)	(0.011)	(0.015)
Children with non-white-collar fathers	6,512	1,173	0.534***	0.210***	2.833***	0.037**	0.039**	-0.029
			(0.167)	(0.064)	(0.863)	(0.017)	(0.017)	(0.023)
Children with low household expenditure	6,462	1,128	0.401**	0.161**	2.343***	0.042**	0.050***	0.000
			(0.173)	(0.065)	(0.888)	(0.017)	(0.017)	(0.024)

Note: Standard errors clustered at the district level are in parentheses. For the list of control variables included in each regression, see Table 2. *, **, and *** indicate statistical significance at the 10%, 5%, and 1% levels, respectively.

Table A5. Summary statistics of the district-level data

Variable	Districts with no junior-high lunch Mean	Std. Dev	Control districts Mean	Std. Dev	
District: mean child height (*z* score)	0.012	0.359	-0.016	0.352	
District: mean child BMI (*z* score)	-0.013	0.352	0.002	0.354	
District: child obesity rate (IOTF BMI 25+)	0.162	0.138	0.157	0.132	
District: child underweight rate (IOTF BMI 18.5-)	0.119	0.115	0.114	0.108	
District: # of participants	81.121	27.520	85.214	26.932	***
District: proportion of age 1–19	0.315	0.068	0.309	0.070	*
District: proportion of age 20–39 (reference)	0.263	0.075	0.259	0.077	
District: proportion of age 40–59	0.277	0.071	0.275	0.072	
District: proportion of age 60+	0.144	0.079	0.156	0.089	***
District: median per-member household expenditure	0.503	0.184	0.569	0.190	***
District: mean household size	4.148	0.584	4.285	0.633	***
District: proportion of white-collar worker (reference)	0.390	0.208	0.375	0.209	
District: proportion of laborer	0.346	0.192	0.344	0.187	
District: proportion of self-employed	0.207	0.153	0.185	0.153	***
District: proportion of agriculture/fisheries/forestry	0.057	0.141	0.097	0.173	***
District: proportion of working women	0.574	0.188	0.608	0.197	***
Prefectural population density (1,000 person/km^2)	0.511	0.434	0.461	0.445	**
Municipal size: 11 largest cities	0.184	0.388	0.033	0.177	***
Municipal size: cities with 150k+ population	0.364	0.482	0.250	0.433	***
Municipal size: cities with 50–150k population	0.222	0.416	0.216	0.411	
Municipal size: cities with 50k- population	0.095	0.294	0.102	0.303	
Municipal size: towns & villages (reference)	0.135	0.342	0.399	0.490	***
Region block: Hokkaido & Tohoku	0.171	0.377	0.184	0.388	
Region block: Kanto	0.080	0.272	0.190	0.392	***
Region block: Chubu (reference)	0.594	0.492	0.485	0.500	***
Region block: Kinki	0.066	0.248	0.017	0.131	***
Region block: Chugoku & Shikoku	0.089	0.285	0.123	0.329	**
Region block: Kyushu & Okinawa	0.252	0.434	0.195	0.396	**
Year	1982.744	5.629	1984.171	5.544	***
Number of districts	473		1,660		

Note: Occupational proportions refer to the proportion of 23- to 54-year-olds in each occupation. The number of districts is smaller than that in the full sample DID regression because 138 districts with less than five children of age 1 to 11 are removed from this sample. *, **, and *** indicate statistically significant differences in means between the control and treatment districts at the 10%, 5%, and 1% levels, respectively.

Table A6. District-level Logit regression of *NoSchoolLunch* dummy

Explanatory variable	Model 1		Model 2	
District: mean child height (*z* score)	0.267	(0.169)	0.100	(0.191)
District: mean child BMI (*z* score)	-0.179	(0.222)	-0.163	(0.252)
District: child obesity rate (IOTF BMI 25+)	0.577	(0.496)	0.364	(0.534)
District: child underweight rate (IOTF BMI 18.5-)	-0.354	(0.635)	0.009	(0.7)
District: # of participants	-0.001	(0.003)	-0.001	(0.003)
District: proportion of ages 1–19	1.238	(1.403)	1.414	(1.528)
District: proportion of ages 40–59	1.286	(0.955)	1.532	(1.053)
District: proportion of ages 60+	1.872*	(1.112)	2.042	(1.253)
District: median per-member household expenditure	-0.573	(0.403)	-0.416	(0.457)
District: mean household size	-0.029	(0.135)	-0.118	(0.156)
District: proportion of laborer	0.293	(0.373)	0.246	(0.397)
District: proportion of self-employed	0.122	(0.434)	0.324	(0.48)
District: proportion of agriculture	-0.309	(0.617)	-0.737	(0.672)
District: proportion of working women	0.619	(0.388)	0.213	(0.442)
Prefectural population density (1,000 person/km^2)	0.143	(0.159)	-3.901**	(1.611)
Municipal size: 11 largest cities	2.867***	(0.27)	3.357***	(0.324)
Municipal size: cities with 150k+ population	1.585***	(0.192)	1.671***	(0.211)
Municipal size: cities with 50–150k population	1.366***	(0.199)	1.388***	(0.22)
Municipal size: cities with 50k- population	1.078***	(0.234)	1.092***	(0.256)
Region block dummies	Yes		No	
Prefecture dummies	No		Yes	
Year dummies	Yes		Yes	
Pseudo R^2	0.153		0.267	
Number of districts	2,133		2,091	

Note: Standard errors are in parentheses. The constant term is included in the model but omitted from the table. The estimated coefficients for year dummies are presented in Table A7. The reference category for the municipal size dummies is towns and villages, that for the age composition variables is the proportion of ages 20 to 39, and that for the occupational composition variables is the proportion of white-collar workers. Districts with less than five respondents of ages 1 to11 are excluded from the sample. Model 2 shows fewer observations than Model 1 because prefectures without variation in the *NoSchoolLunch* dummy are omitted. *, **, and *** indicate statistical significance at the 10%, 5%, and 1% levels, respectively.

Table A7. The estimated year effects from Logit regression of *NoSchoolLunch*

Variable	Model1 (N=2,133)		Model2 (N=2,091)	
Year 1976	0.057	(0.298)	0.090	(0.334)
Year 1977	0.095	(0.318)	0.214	(0.377)
Year 1978	-0.265	(0.317)	-0.165	(0.368)
Year 1979	-0.244	(0.333)	-0.170	(0.365)
Year 1980	-0.417	(0.318)	-0.353	(0.365)
Year 1981	-0.317	(0.335)	-0.329	(0.385)
Year 1982	-0.536	(0.333)	-0.336	(0.374)
Year 1983	-1.135***	(0.382)	-1.118**	(0.437)
Year 1984	-0.851**	(0.345)	-0.742*	(0.392)
Year 1985	-0.713**	(0.344)	-0.641	(0.401)
Year 1986	-1.143***	(0.339)	-1.052***	(0.391)
Year 1987	-0.996***	(0.351)	-0.741*	(0.403)
Year 1988	-0.735**	(0.334)	-0.633	(0.393)
Year 1989	-1.444***	(0.416)	-1.399***	(0.466)
Year 1990	-0.979***	(0.365)	-0.978**	(0.438)
Year 1991	-0.759**	(0.358)	-0.460	(0.409)
Year 1992	-0.835**	(0.37)	-0.723*	(0.433)
Year 1993	-1.330***	(0.416)	-1.148**	(0.464)
Year 1994	-1.213***	(0.445)	-1.040**	(0.522)
Region block dummies	Yes		No	
Prefecture dummies	No		Yes	

Note: This table presents the year effects estimated in the *NoSchoolLunch* Logit regression, which are not reported in Table A6. The reference year is 1975. Standard errors are in parentheses. *, **, and *** indicate statistical significance at the 10%, 5%, and 1% levels, respectively.

Table A8. Effects of no school lunch: Different thresholds for low per-member household expenditure.

Panel (a): DID

Sample	# children	# districts	BMI	BMI z-score	POW	Obesity (IOTF)	Obesity (POW)	Underweight (IOTF)
Below 40th percentile	6,442	1,188	0.383**	0.152**	2.447***	0.053***	0.063***	0.012
			(0.178)	(0.067)	(0.901)	(0.018)	(0.017)	(0.024)
Below median (reshown)	8,389	1,455	0.332**	0.126**	2.067***	0.046***	0.053***	0.001
			(0.153)	(0.057)	(0.793)	(0.015)	(0.015)	(0.022)
Below 60th percentile	10,309	1,684	0.351***	0.138***	1.883***	0.040***	0.045***	(0.015)
			(0.130)	(0.049)	(0.673)	(0.013)	(0.013)	(0.019)
Below 70th percentile	12,239	1,850	0.204*	0.074*	1.155*	0.023*	0.026**	(0.012)
			(0.115)	(0.044)	(0.599)	(0.012)	(0.012)	(0.017)

Panel (b): DID-IPTW

Sample	# children	# districts	BMI	BMI z-score	POW	Obesity (IOTF)	Obesity (POW)	Underweight (IOTF)
Below 40th percentile	4,568	861	0.498***	0.214***	2.982***	0.057***	0.063***	0.006
			(0.186)	(0.070)	(0.953)	(0.020)	(0.019)	(0.030)
Below median (reshown)	6,462	1,128	0.410**	0.168***	2.440***	0.052***	0.056***	0.001
			(0.165)	(0.061)	(0.843)	(0.017)	(0.017)	(0.025)
Below 60th percentile	7,897	1,295	0.434***	0.176***	2.330***	0.045***	0.049***	-0.017
			(0.145)	(0.055)	(0.739)	(0.015)	(0.015)	(0.022)
Below 70th percentile	9,298	1,402	0.258*	0.105**	1.425**	0.022	0.027**	-0.022
			(0.134)	(0.050)	(0.682)	(0.014)	(0.014)	(0.020)

Note: The percentiles are for the per-member household expenditure. The estimates for children below median household expenditure are reposted from Table 3. Standard errors clustered at the district level are in parentheses. For the list of control variables included in each regression, see Table 2. *, **, and *** indicate statistical significance at the 10%, 5%, and 1% levels, respectively.

Table A9. The falsification test: regression analysis of height

Panel (a): Base DID

Sample	# children	# districts	Height	Height *z*-score
Full sample	18,305	2,271	0.002	0.002
			(0.214)	(0.033)
Children with non-white-collar fathers	9,182	1,654	0.416	0.067
			(0.291)	(0.046)
Children with low household expenditure	8,389	1,455	-0.027	-0.010
			(0.340)	(0.052)

Panel (b): DID-IPTW

Sample	# children	# districts	Height	Height *z*-score
Full sample	13,985	1,741	-0.126	-0.018
			(0.259)	(0.040)
Children with non-white-collar fathers	6,512	1,173	0.382	0.058
			(0.336)	(0.054)
Children with low household expenditure	6,462	1,128	-0.124	-0.024
			(0.377)	(0.058)

Note: Standard errors clustered at the district level are in parentheses. For the list of control variables included in each regression, see Table 2. *, **, and *** indicate statistical significance at the 10%, 5%, and 1% levels, respectively.
